# Salivary biomarkers in breast cancer diagnosis: A systematic review and diagnostic meta‐analysis

**DOI:** 10.1002/cam4.4640

**Published:** 2022-03-22

**Authors:** Maryam Koopaie, Sajad Kolahdooz, Mahnaz Fatahzadeh, Soheila Manifar

**Affiliations:** ^1^ Tehran University of Medical Sciences Tehran Iran; ^2^ USERN, Tehran University of Medical Sciences Tehran Iran; ^3^ Department of Diagnostic Sciences Rutgers School of Dental Medicine Newark New Jersey USA; ^4^ Cancer Research Center, Cancer Institute of Iran Tehran Iran

**Keywords:** biomarker, breast cancer, diagnosis, meta‐analysis, saliva

## Abstract

**Background:**

Salivary diagnostics and their utility as a nonaggressive approach for breast cancer diagnosis have been extensively studied in recent years. This meta‐analysis assesses the diagnostic value of salivary biomarkers in differentiating between patients with breast cancer and controls.

**Methods:**

We conducted a meta‐analysis and systematic review of studies related to salivary diagnostics published in PubMed, EMBASE, Scopus, Ovid, Science Direct, Web of Science (WOS), and Google Scholar. The articles were chosen utilizing inclusion and exclusion criteria, as well as assessing their quality. Specificity and sensitivity, along with negative and positive likelihood ratios (NLR and PLR) and diagnostic odds ratio (DOR), were calculated based on random‐ or fixed‐effects model. Area under the curve (AUC) and summary receiver‐operating characteristic (SROC) were plotted and evaluated, and Fagan's Nomogram was evaluated for clinical utility.

**Results:**

Our systematic review and meta‐analysis included 14 papers containing 121 study units with 8639 adult subjects (4149 breast cancer patients and 4490 controls without cancer). The pooled specificity and sensitivity were 0.727 (95% CI: 0.713–0.740) and 0.717 (95% CI: 0.703–0.730), respectively. The pooled NLR and PLR were 0.396 (95% CI: 0.364–0.432) and 2.597 (95% CI: 2.389–2.824), respectively. The pooled DOR was 7.837 (95% CI: 6.624–9.277), with the AUC equal to 0.801. The Fagan's nomogram showed post‐test probabilities of 28% and 72% for negative and positive outcomes, respectively. We also conducted subgroup analyses to determine specificity, sensitivity, DOR, PLR, and NLR based on the mean age of patients (≤52 or >52 years old), saliva type (stimulated and unstimulated saliva), biomarker measurement method (mass spectrometry [MS] and non‐MS measurement methods), sample size (≤55 or >55), biomarker type (proteomics, metabolomics, transcriptomics and proteomics, and reagent‐free biophotonic), and nations.

**Conclusion:**

Saliva, as a noninvasive biomarker, has the potential to accurately differentiate breast cancer patients from healthy controls.

## Graphical abstract

Our meta‐analysis showed that saliva might be a promising, efficient, and noninvasive biomarker with the potential to differentiate between patients with and without breast cancer. Subgroup analyses were done to determine sensitivity, specificity, PLR, NLR, and DOR based on the mean age of patients, saliva type (stimulated and unstimulated saliva), biomarker measurement method (mass spectrometry [MS] and non‐MS measurement methods), sample size, biomarker type (proteomics, metabolomics, transcriptomics and proteomics, and reagent‐free biophotonic), and nations. Unstimulated saliva has a more acceptable diagnostic value compared to stimulated saliva in breast cancer.

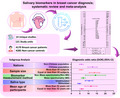

## 1 INTRODUCTION

Breast cancer (BC) is the most frequent malignancy in females globally and the major cause of cancer death in women.^1^, ^2^ The incidence of BC is rising rapidly in the developed countries.^3^, ^4^ Early detection of BC not only improves therapeutic outcomes, but also positively impacts the psychological, economic, and social complications of this malignant disease.^5^, ^6^ Unfortunately, in many countries, women face a multitude of challenges such as social, economic, and cultural barriers to the early detection of BC.^7^, ^8^ Therefore, the search for cost‐effective, noninvasive diagnostics for BC has prompted extensive investigations aimed at identifying liquid biomarkers and analyzing their efficacy for this purpose.^9^, ^10^ In particular, the liquid biopsy approach for the detection of tumor‐derived biomarkers (cellular, molecular, and genomic) in the blood and other body fluids such as saliva has attracted much attention for diagnostic and prognostic evaluation of various cancers, including BC.^11^, ^12^

The utility of saliva for cancer diagnosis prior to the development of clinical, histological, and radiological signs could offer a promising approach for developing personalized medicine strategies.^15^ Saliva, a biofluid which mirrors the body's health, has been used to screen, diagnose, and follow BC in many recent studies.^13^, ^14^, ^15^, ^16^, ^17^ These investigations have proposed a variety of salivary biomarkers, including proteome, metabolome, transcriptome, and reagent‐free biophotonic.^12^, ^18^, ^19^, ^20^, ^21^, ^22^, ^23^, ^24^, ^25^, ^26^, ^27^, ^28^, ^29^, ^30^, ^31^, ^32^, ^33^ In fact, intense research in this area has led to the nomenclature of “salivaomics” which describes saliva‐based diagnostics.^12^, ^34^ Using saliva for diagnosis has many advantages, including easy collection, minimal training requirement for staff, rapid sampling, hassle‐free storage, simplicity of transportation, less sensitivity to clotting, and fewer risks for the health staff.^15^, ^35^, ^36^, ^37^ Despite these advantages, the presence or concentration of biomarkers in saliva may differ from the other body bio‐fluids,^38^, ^39^ and it is critical to determine which salivary biomarkers provide acceptable sensitivity and specificity for the diagnosis of BC. Although studies have examined salivary biomarkers in distant malignancies^33^, ^40^, ^41^; the pathophysiologic effect of these cancers on salivary profiles remains unclear. There is some evidence that cells of salivary glands and mammary glands are pathologically and functionally similar.^42^, ^43^, ^44^ Also, salivary gland cells secrete exosome‐like microvesicles, which encapsulate both proteins and mRNAs and can be detected in saliva.^43^

Several studies have compared salivary protein levels in BC patients and healthy controls using various methods.^45^, ^46^, ^47^ Efforts have also been undertaken to assess the efficacy of salivary C‐erb‐B2 (HER2) levels in BC patients.^48^, ^49^ In addition, detection of sialic acid (SA) and the impact of disease stage and chemotherapeutics on the levels of SA and sialo‐glycomic in saliva from BC patients have been assessed.^26^, ^50^, ^51^ Moreover, salivary levels of lectins, polyamines, and N‐acetylated in BC patients have been examined in multiple investigations.^24^, ^25^, ^52^, ^53^ In their review study, Porto‐Mascarenhas et al. concluded that salivary biomarkers might be more readily detectable in advanced stages compared to early stages of BC.^54^ Recent studies of metabolites in saliva have revealed that metabolites can differentiate BC patients from healthy individuals.^23^, ^24^, ^29^ Researchers postulate that salivary levels of some mRNAs could have high diagnostic accuracy for BC.^17^, ^20^ Despite the depth and breadth of salivaomics and the promising outcome for salivary diagnostics replicated in numerous studies, these investigations differ in many respects, including sample size, saliva type (stimulated or unstimulated saliva), saliva collection method, measurement method of salivary biomarkers, biomarker type, and the methodology for detection and measurement of biomarkers. These variations in study design could potentially affect the diagnostic accuracy of salivary biomarkers, and a comprehensive review of the proposed salivary biomarkers as a reliable diagnostic source for BC diagnosis and future studies in this field is highly warranted. To the best of our knowledge, no systematic review has been conducted in this field. Therefore, we aim to conduct a meta‐analysis and systematic review to explore the diagnostic value of saliva for BC detection.

## 2 METHODS

This study was conducted in accordance with the Preferred Reporting Items for Systematic Reviews and Meta‐Analyses (PRISMA) guidelines.^55^ We aim to answer the following question: “What are the diagnostic values of salivary biomarkers in BC patients versus controls without BC?” And “can biomarkers in saliva serve a role in BC diagnosis?”

### 2.1 Search strategy

A comprehensive approach and strategy were implemented to search the PubMed, EMBASE, Scopus, Ovid, Science Direct, Web of Science (WOS), and Google Scholar bibliographic databases. The keywords used for the search included “breast cancer,” “diagnosis,” “saliva,” and “salivary biomarker.” We also identified references cited in the eligible articles that could have been unintentionally omitted during the search. All review‐related work was performed in February 2021 and updated in August 2021. Using the reference manager program (EndNote X9.0; Thomson Reuters, USA), all references were managed, and redundant sources were removed. Articles were screened for the eligibility criteria through the analysis of their titles as well as abstracts by each author independently. Selected articles were scrutinized thoroughly to confirm eligibility using the Quality Assessment of Diagnostic Accuracy Studies 2 checklist (QUADAS‐2).

### 2.2 Eligibility criteria

#### 2.2.1 Inclusion criteria

The following were defined as criteria for inclusion: (1) Diagnostic and screening studies using salivary biomarkers for breast malignancies. (2) Studies with BC patients and noncancerous controls. (3) Studies with sufficient data to obtain true positive (TP), false positive (FP), true negative (TN), and false‐negative (FN) values.

#### 2.2.2 Exclusion criteria

Based on the exclusion criteria, the following studies were excluded: (1) Case reports, letters, personal opinions, reviews, book chapters, short communications, conference abstracts, and patents; (2) Duplicate publications; (3) In‐vivo and also in‐vitro researches that reported an association between saliva and BC; (4) Studies with no existing data or incomplete information.

Following that, the authors individually reviewed the entire content of eligible studies to determine appropriateness. Disagreements among the authors were discussed until the consensus was reached.

### 2.3 Data extraction

Three authors who extracted data individually from each eligible study. The extracted data included; publication year, first author, country, number of controls and cases, study design, control group type: healthy controls and non‐cancer controls (benign or mixed), biomarker type (proteomics, metabolomics, microbiome, and transcriptomic), type of saliva sample (unstimulated or stimulated), age of participants, cut‐off point, area under curve (AUC) of receiver characteristic operator (ROC), methodology, and stage of BC.

### 2.4 Quality assessment

The quality of selected papers was appraised separately by authors using QUADAS‐2.^56^ If there were disagreements between evaluators, they strived for consensus through discussion. Utilizing the QUADAS‐2 checklist, selected studies were assessed in four main areas: patient selection, index test, reference standard, as well as flow and timing for bias risk. Patient selection, index test, and reference standard, were also evaluated for applicability. Bias risk and applicability were rated “low,” “high,” and “unclear” for each domain. Articles were also categorized based on quality into low, medium, and high quality.

### 2.5 Statistical analysis

Meta‐Disc v.1.4 software (Madrid, Spain), Comprehensive Meta‐Analysis (CMA) v.3.3.070 (Biostat, USA), STATA v.15.0 (https://www.stata.com), and MetaDTA v.2.01 (https://crsu.shinyapps.io/dta_ma/) were used to perform statistical analysis. Diagnostic value of salivary biomarkers for BC was assessed by the pooled specificity and sensitivity, along with negative and positive likelihood ratios (NLR and PLR), as well as diagnostic odds ratio (DOR) with 95% confidence interval (CI), and AUC of hierarchical ROC (HROC). AUC of summary ROC (SROC) was used to evaluate the overall diagnostic performance of salivary biomarkers. For this purpose, the bivariate generalized linear model was used to extract TP, FP, TN, and FN in individual studies. For obtaining pooled specificity and sensitivity, along with pooled NLR and PLR and pooled DOR, random‐effects model was used to combine studies. For statistical analysis between sensitivity and specificity, Spearman correlation was applied. Fagan's nomogram was plotted to assess the utility of salivary biomarkers for clinical diagnosis of BC. Evaluation of heterogeneity between studies was done by using tau‐squared (τ^2^), I‐square (I^2^) index, and Cochran's Q. Statistically significant p‐value (p) was considered lower than 0.05. Funnel plot was applied for visual investigation of publication bias, and for assessment of funnel plot asymmetry, Egger's test was used.^57^ Subgroup analyses were conducted to determine the sensitivity, specificity, PLR, NLR, DOR reports, and heterogeneity. Meta‐regression was performed to define the effect of saliva type, biomarker type, sample size, and mean age of patients on heterogeneity. A graphical quality assessment provided SROC plot was utilized to illustrate the findings of individual studies in the meta‐analysis with quality indicators assessed using QUADAS‐2.^58^ During the quality assessment of eligible studies, we used red, green, and gray markings to flag studies with high, low, and uncertain risk of bias, respectively, using the glyph system.

## 3 RESULTS

### 3.1 Study selection

**FIGURE 1:**
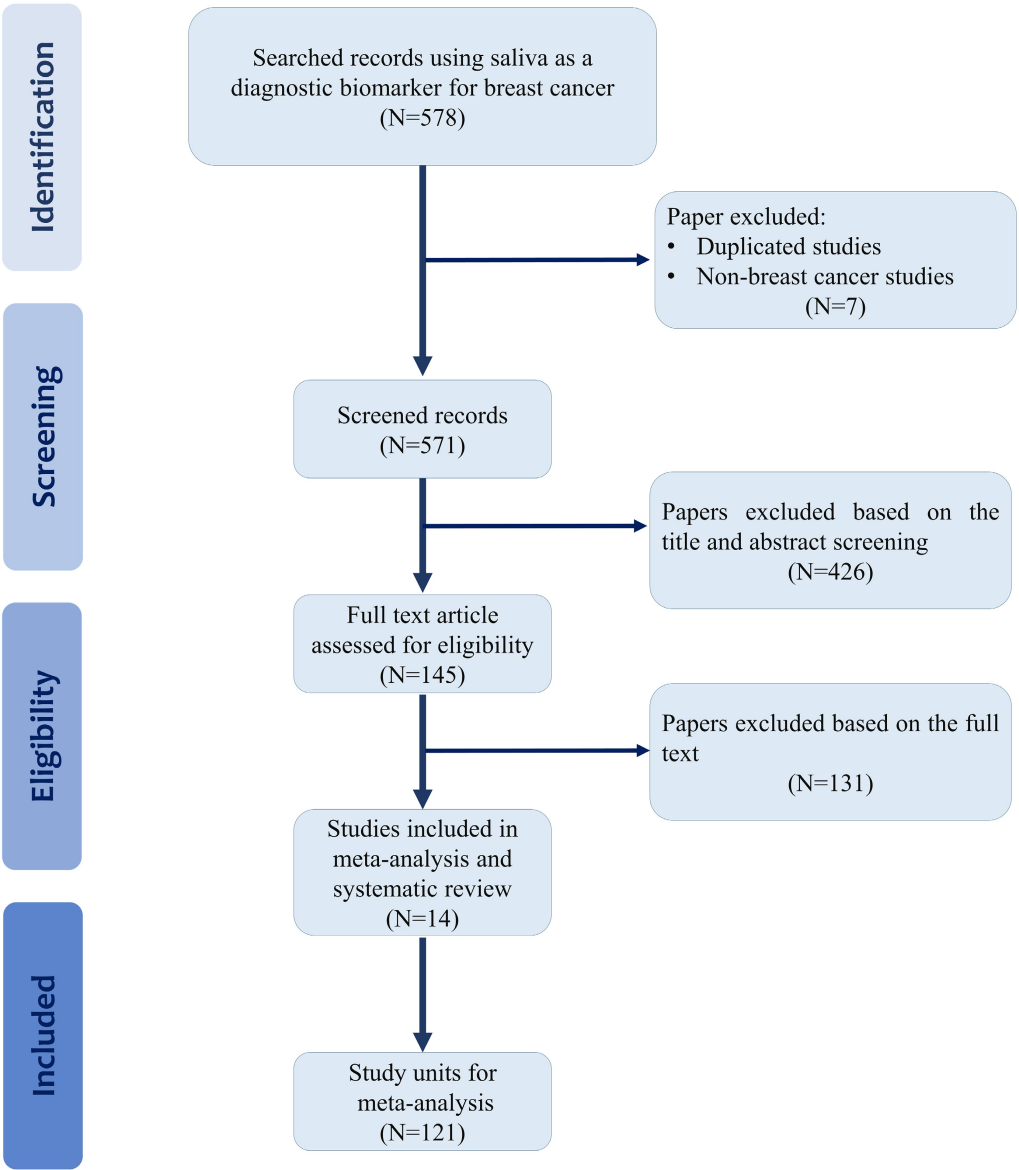
Flowchart illustrating the criteria and process for the literature search

Five hundred and seventy‐eight citations were retrieved through the systematic search (Figure 1). Following an evaluation of the article titles and abstracts, 145 articles were approved for review of full text, and 131 articles were excluded based on the exclusion criteria. The search process resulted in 14 articles meeting the inclusion criteria and being eligible for this study.^18^, ^19^, ^20^, ^21^, ^22^, ^23^, ^24^, ^25^, ^26^, ^27^, ^28^, ^29^, ^30^, ^31^

### 3.2 Literature search and study characteristics

Fourteen studies published since 1998 and related to the use of salivary biomarkers in the diagnosis and screening of BC were evaluated. A summary of salivary biomarkers used in the eligible studies is provided in Table 1.

**TABLE 1:** Characteristics of eligible studies

	Author (Year)	Saliva type	Country	Method of measurement	Biomarker type	Sample Size			Biomarker
						Case	Control	Benign	
1	Streckfus et al.^18^	Stimulated	USA	ELISA	Proteomics	30	57	44	CA15‐3, HER2
2	Brooks et al.^19^	Unstimulated	USA	ELISA	Proteomics	49	49	‐	VEGF, EGF, VEGF + EGF, CEA
3	Zhang et al.^20^	Unstimulated	USA	RT‐qPCR	Transcriptomics and Proteomics	30	63	‐	One protein and eight mRNAs
4	Cheng et al.^21^	Unstimulated	China	UPLC–MS	Proteomics	17	28	‐	15 free amino acid profile
5	Wood et al.^22^	Stimulated	USA	Electrophoresis & Western blot	Proteomics	16	16	‐	Total protein
6	Zhong et al.^23^	Unstimulated	China	HILIC‐UPLC–MS	Metabolomics	30	25	‐	18 metabolites
7	Takayama et al.^24^	Unstimulated	Japan	UPLC‐ESI‐MS	Metabolomics	111	61	‐	13 polyamines
8	Liu et al.^25^	Unstimulated	China	Blotting analysis	Proteomics	27	13	21	Two lectins (BS‐I and NPA)
9	Hernández‐Arteaga et al.^26^	Unstimulated	Mexico	SERS	Proteomics	35		129	Sialic acid
10	Farahani et al.^27^	Unstimulated	Iran	ELISA	Proteomics	30	30	‐	CA15‐3, CEA, estradiol, vaspin, obestatin
11	Ferreira et al.^28^	Stimulated	Brzazil	ATR‐FTIR Spectroscopy	Reagent‐free biophotonic	10	10	10	ATR‐FTIR Spectroscopy
12	Assad et al.^29^	Stimulated	Brzazil	LC/MS	Metabolomics	23	35	‐	31 metabolomics including seven oligopeptides and six glycerophospholipids
13	Bel'skaya et al.^30^	Unstimulated	Russia	ELISA	Metabolomics	43	39	32	L‐arginine metabolism, NO, arginase/NO, Cytokines (IL‐2.4,6,10,18)
14	López‐Jornet et al.^31^	Unstimulated	Spain	Total antioxidant capacity and ferric reducing ability of plasma	Proteomics	91	60	‐	CA125, sFas, Combination of CA125 and sFas

Abbreviations: ATR‐FTIR Spectroscopy, Attenuated total reflection‐fourier transform infrared Spectroscopy; ELISA, Enzyme‐linked immunosorbent assay; HILIC‐UPLC–MS, Hydrophilic interaction chromatography‐Ultra‐performance liquid chromatography‐mass spectrometry; LC/MS, Liquid chromatography/mass spectrometry; RT‐qPCR, Reverse transcription quantitative polymerase chain reaction; SERS, Surface enhanced Raman spectroscopy.

### 3.3 Study characteristics

One hundred twenty one study units were included in this study. Considering the saliva‐omics classification, there were eight proteomics studies, four metabolic studies, one transcriptomics and proteomics study, and one reagent‐free biophotonic study. These articles were published between February 1, 2000 and September 1, 2021, based on research in eight countries: four studies performed in the United States, three in China, two in Brazil, one in Japan, and one in Russia. One investigation was performed in Spain, one in Iran, and one in Mexico. In 10 out of 14 studies, the saliva sample used was of unstimulated type and in the remaining four studies, stimulated saliva was utilized. Two studies analyzed a single salivary biomarker, and 12 studies evaluated multiple biomarkers or combinations of them. This meta‐analysis was based on 14 papers and 121 study units, which included a total sample size of 8639 subjects (4149 BC patients and 4490 non‐cancer controls). The information extracted from eligible studies pertained to 152 infiltrating ductal carcinoma, 84 ductal carcinoma, 22 ductal carcinoma in situ, three infiltrating lobular carcinoma, one mucinous carcinoma, and 12 other types of breast cancer.^18^, ^19^, ^20^, ^21^, ^22^, ^23^, ^24^, ^25^, ^26^, ^27^, ^28^, ^29^, ^30^, ^31^ For the studies included in our review, 349 patients had stages 1 and 2, while 102 patients had stages 3 and 4 BC.^18^, ^19^, ^20^, ^28^, ^29^, ^30^, ^31^

### 3.4 Quality assessment of included studies

**FIGURE 2:**
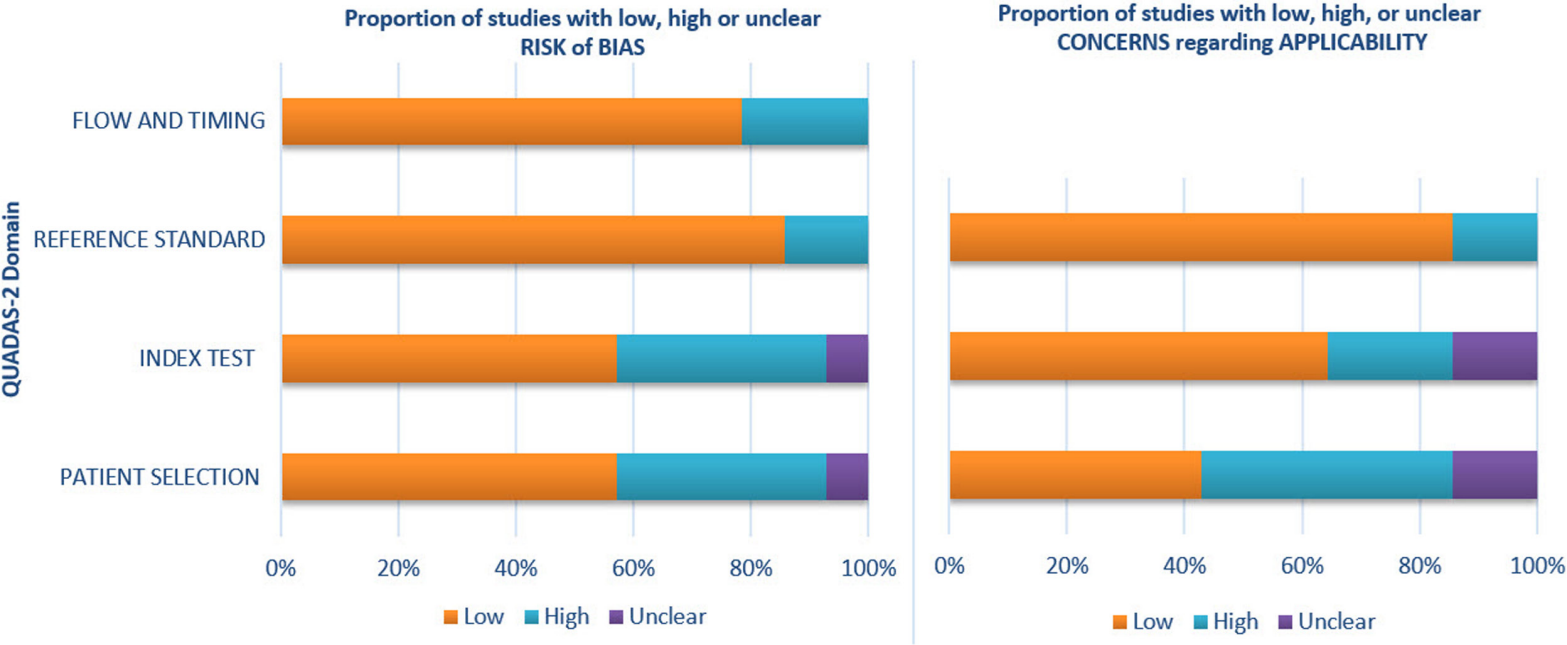
Overall results of quality assessments for included studies using the QUADAS‐2 tool

In the majority of studies categorized as “high risk” for bias, the concern was related to the patient selection and the index test (Figure 2). In the majority of studies classified as “high risk” for applicability, the concern was primarily related to the patient selection (Figure 2). In the majority of studies classified as “low risk” for bias and applicability, the classification reflected the reference standard (Figure 2). In the majority of studies classified as “unclear” for either bias risk or applicability, the concern pertained to the patient selection and the index test (Supplementary file, Table S1).

### 3.5 Test of heterogeneity

Heterogeneity due to threshold was assessed by Spearman's correlation coefficient (SCC). The SCC was 0.034 (*p* = 0.713) (log [(TP rate)/(1‐(TP rate))] vs. log [(FP rate)/(1‐(FP rate))]) suggesting lack of diagnostic threshold effect (Table 2). The I^2^ heterogeneity of specificity, sensitivity, NLR, PLR, and DOR were 66.1% (*p* < 0.0001), 62.2% (*p* < 0.0001), 54.3% (*p* < 0.0001), 56.5% (*p* < 0.0001), and 56.2% (*p* < 0.0001), respectively. These results could indicate heterogeneity among the studies included in our analysis.

**TABLE 2:** Moses' model (D = α + βS) for diagnostic threshold (inverse variance and study size) of BC by salivary biomarkers in breast cancer diagnosis

Variation	Coefficient	Standard error	T	*p*
α	2.057	0.086	24.043	0.0000
β	0.046	0.086	0.534	0.5942
Inverse Variance. τ^2^ = 0.4346 (5 iterations lead to convergence).				
α	2.322	0.100	23.119	0.0000
β	0.050	0.089	0.564	0.5739
Study Size. τ^2^ = 1.2016 (3 iterations lead to convergence).				

### 3.6 Diagnostic values of salivary biomarkers

**FIGURE 3:**
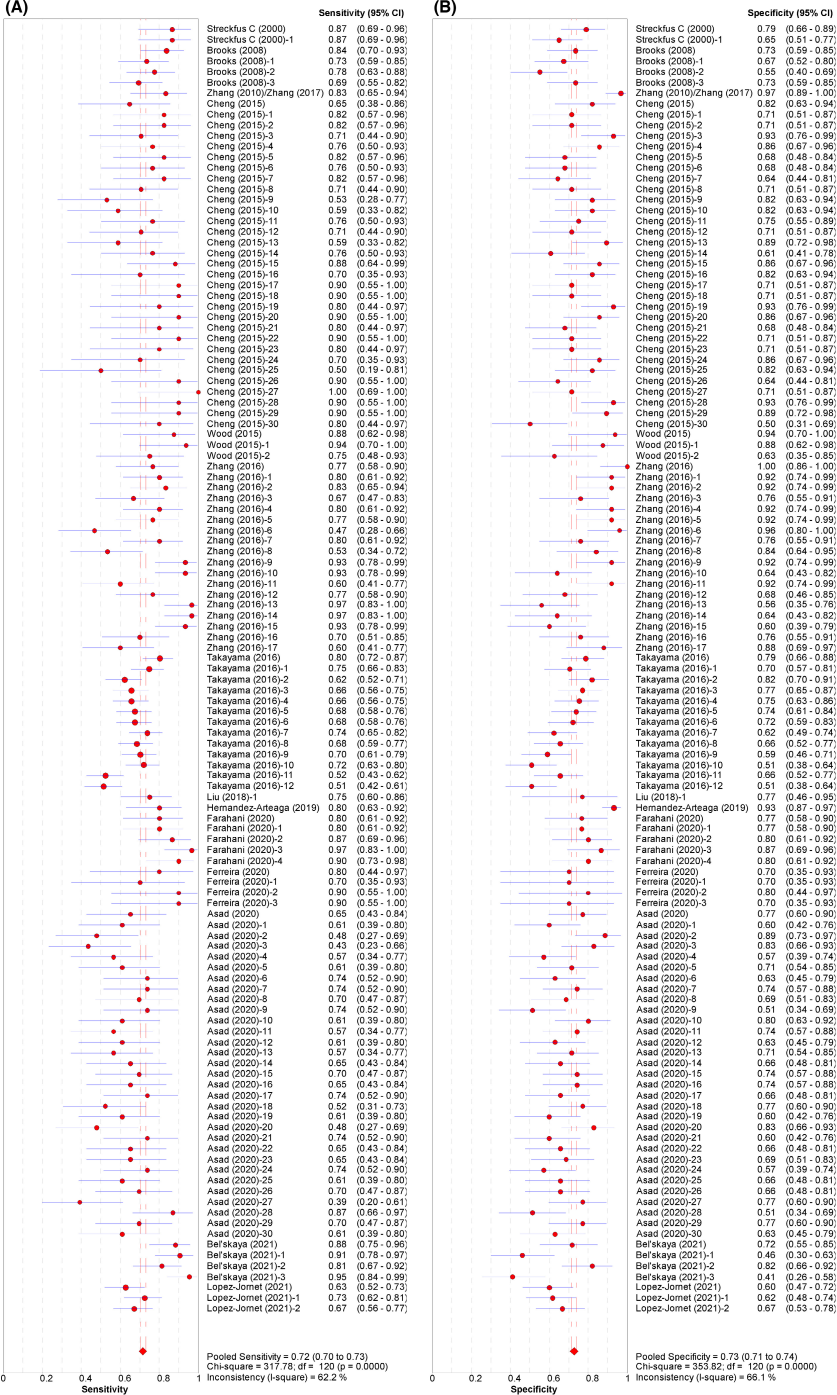
Paired forest plot of (A) sensitivity and (B) specificity for the salivary diagnosis of BC (95% CI)

**FIGURE 4:**
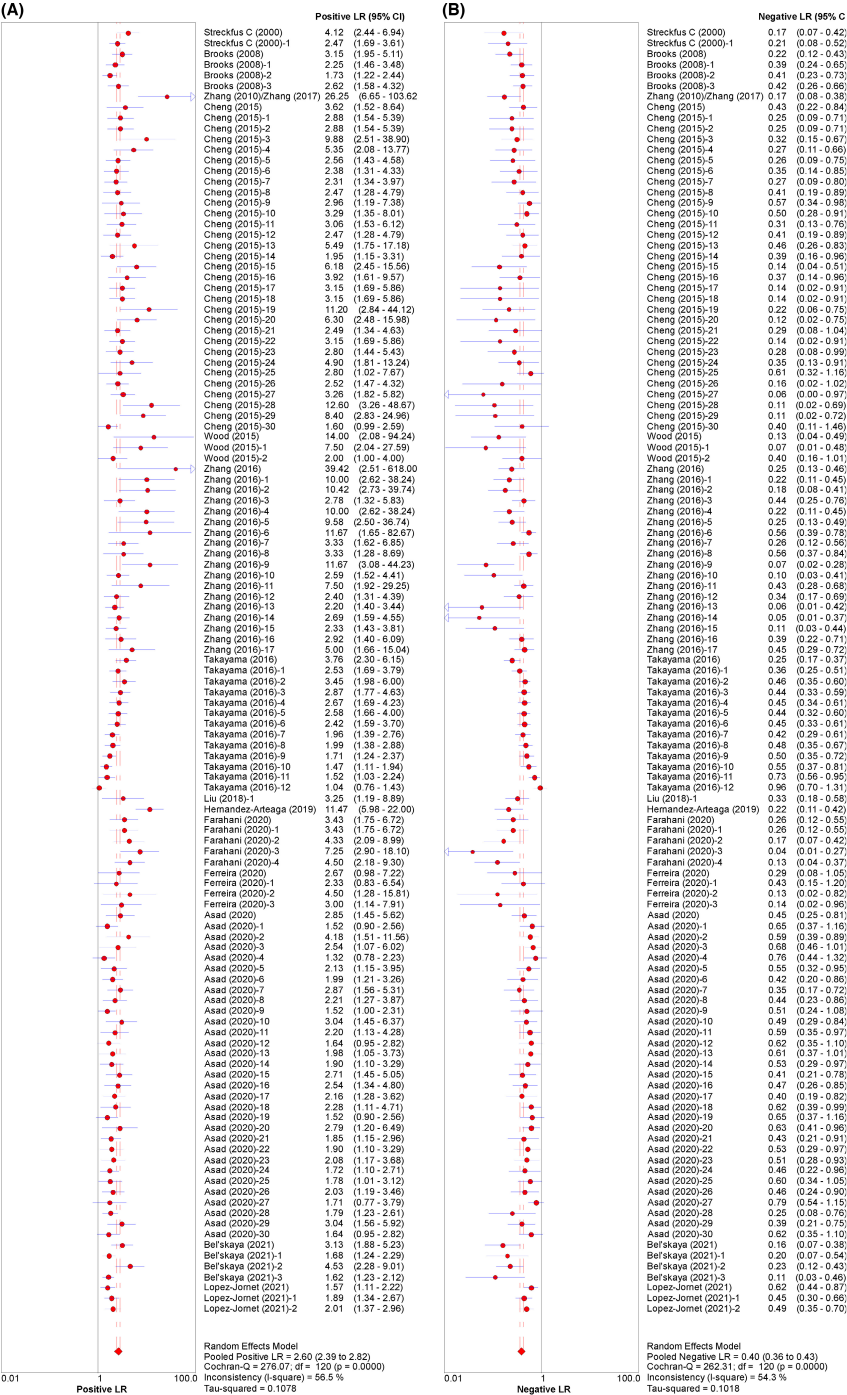
Paired forest plot of (A) PLR and (B) NLR for salivary diagnosis of BC (95% CI)

**FIGURE 5:**
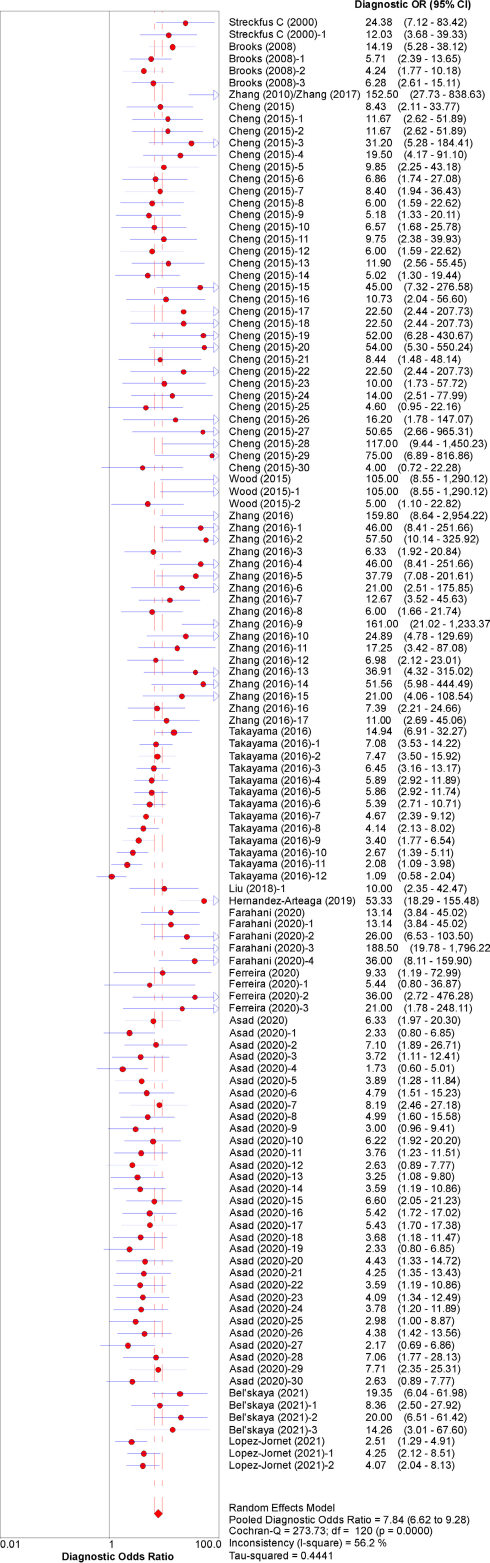
Forest plot of DOR for the salivary diagnosis of BC (95% CI)

**FIGURE 6:**
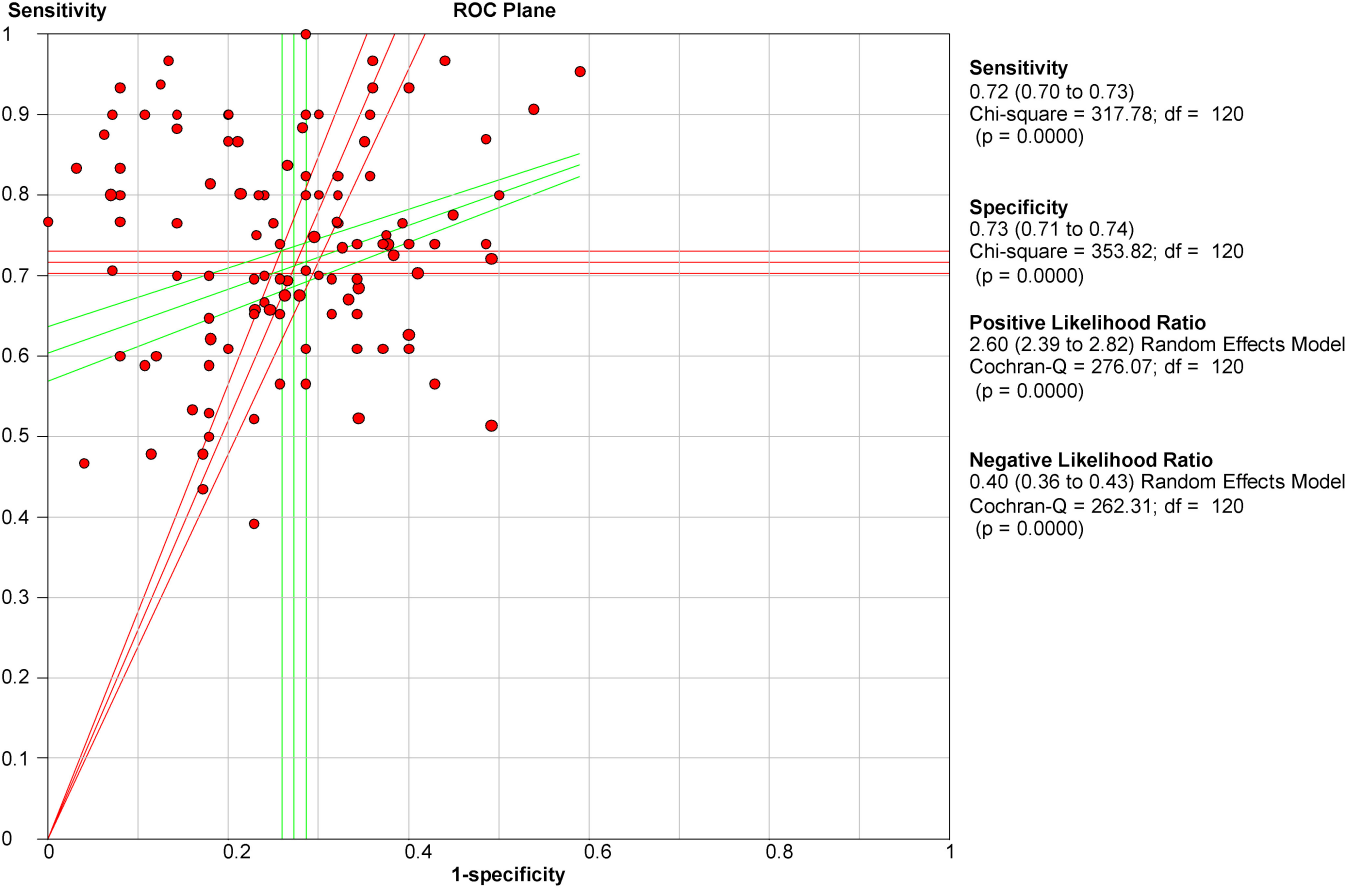
ROC plane curve for the salivary diagnosis of BC revealed the threshold effects between the pooled sensitivity and 1‐specificity

**FIGURE 7:**
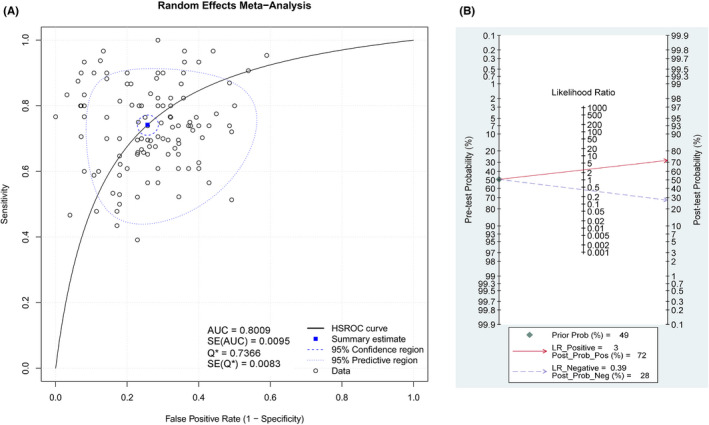
(A) HSROC curves and (B) Fagan's nomogram for the salivary diagnosis of BC

The pooled sensitivity and specificity were 0.717 (95% CI: 0.703–0.730) and 0.727 (95% CI: 0.713–0.740), respectively (Figure 3A,B). The pooled PLR and NLR were 2.597 (95% CI: 2.389–2.824) and 0.396 (95% CI: 0.364–0.432), respectively (Figure 4A,B). The pooled DOR was 7.837 (95% CI: 6.621–9.277) (Figure 5), with the AUC equal to 0.801. The ROC plane of sensitivity, specificity, and hierarchical SROC (HSROC) curves for diagnosis of BC are shown in Figures 6 and 7‐A, respectively.

The ROC plane of sensitivity in Figure 6 explores the threshold effects between the pooled sensitivity and specificity. The HSROC was applied to summarize salivary biomarkers' overall diagnostic performance with 95% CI (Figure 7A). Fagan's nomogram showed that with the pre‐test probability of 49%, the post‐test probability reached 72% and 28% for the positive and negative tests, respectively (Figure 7B). These results confirmed the high diagnostic efficiency of salivary biomarkers in the diagnosis of BC.

### 3.7 Subgroup analysis

Subgroup analyses were carried out in accordance with the mean age of patients (≤52, >52), saliva type (stimulated and unstimulated), biomarker measurement method (mass spectrometry and non‐mass spectrometry), sample size (≤55, >55), biomarker type (proteomics, metabolomics, transcriptomics, proteomics, and reagent‐free biophotonic), and type of control (healthy controls and non‐cancer controls (benign or mixed)). The diagnostic threshold based on the SCC for each subgroup was analyzed (Supplementary file, Table S2).

#### 3.7.1 Mean age of patients

The sensitivity and specificity of salivary biomarkers for the BC diagnosis in patients with the mean age ≤ 52 years were 0.711 (95% CI: 0.689–0.733) and 0.708 (95% CI: 0.689–0.726), respectively. The PLR, NLR, and DOR of the salivary biomarker in patients ≤52 years were 2.348 (95% CI: 2.157–2.557), 0.446 (95% CI: 0.404–0.492), and 6.092 (95% CI: 5.122–7.246), respectively (Supplementary file, Figure S1–S3). The pooled sensitivity and pooled specificity of salivary biomarkers for patients >52 years were 0.720 (95% CI: 0.702–0.738) and 0.752 (95% CI: 0.732–0.772), respectively. The PLR, NLR, and DOR of salivary biomarkers for patients >52 years were 3.068 (95% CI: 2.595–3.627), 0.345 (95% CI: 0.298–0.399), and 11.212 (95% CI: 8.175–15.376), respectively (Supplementary file, Figure S4–S6). The AUC for patients ≤52 years old was 0.770, and AUC for patients >52 years old was 0.8402 (Supplementary file, Figures S3 & S6).

#### 3.7.2 Stimulated and unstimulated saliva

Subgroup analysis was also undertaken in terms of saliva type (stimulated and unstimulated). The sensitivity, specificity, PLR, NLR, and DOR of stimulated saliva for BC diagnosis were 0.670 (95% CI: 0.638–0.702), 0.697 (95% CI: 0.671–0.722), 2.094 (95% CI: 1.906–2.301), 0.516 (95% CI: 0.463–0.576), and 4.628 (95% CI: 3.781–5.663), respectively (Supplementary file, Figure S7). The AUC of stimulated saliva was 0.739 (Supplementary file, Figure S8). The sensitivity, specificity, PLR, NLR, and DOR of unstimulated saliva for diagnosis of BC were 0.729 (95% CI: 0.713–0.744), 0.740 (95% CI: 0.724–0.755), 2.912 (95% CI: 2.600–3.261), 0.350 (95% CI: 0.313–0.391), and 10.300 (95% CI: 8.241–12.874), respectively (Supplementary file, Figure S9). The AUC of unstimulated saliva was 0.830 (Supplementary file, Figure S10).

#### 3.7.3 Mass spectrometry (MS) and non‐MS measurement methods

**FIGURE 8:**
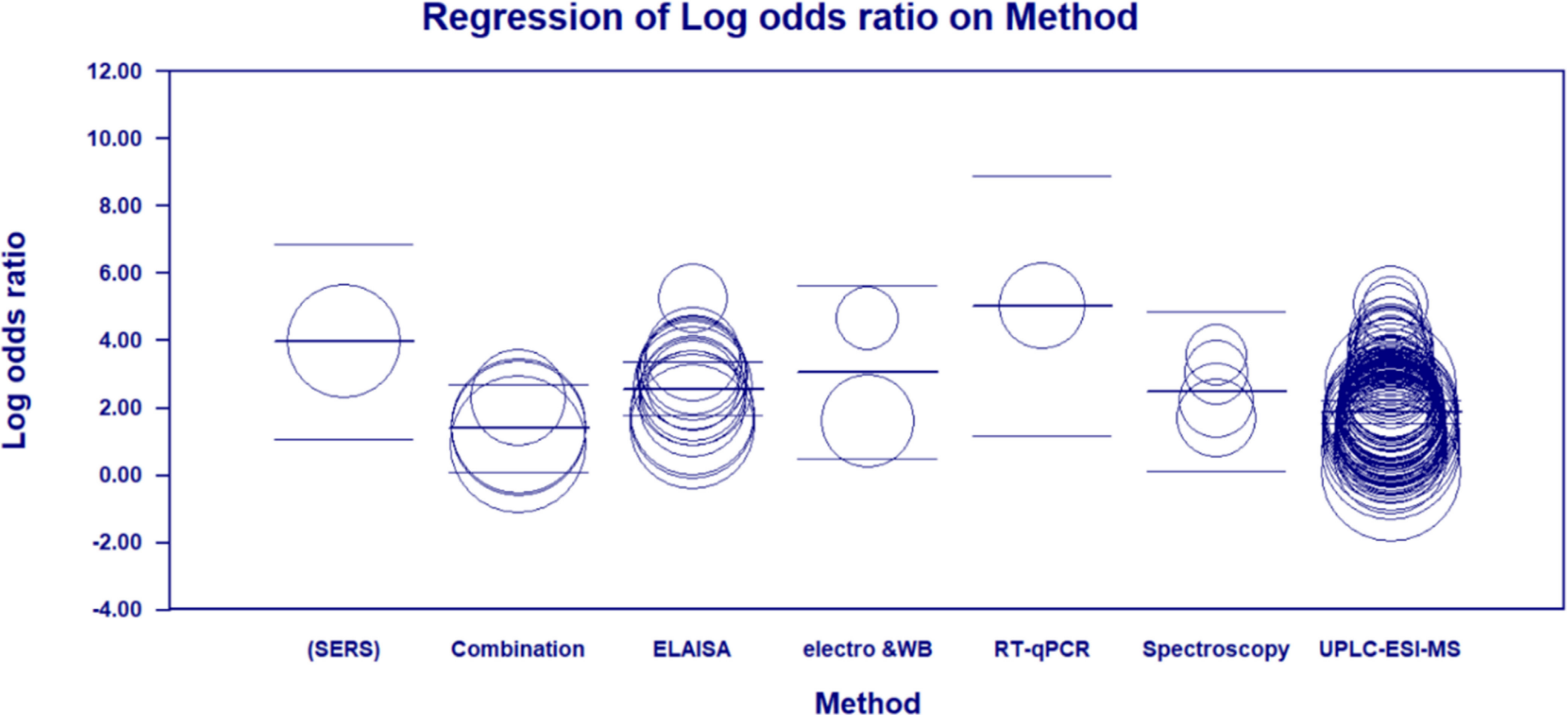
Scatter plot of meta‐regression based on the methodology used to measure salivary biomarkers

We conducted additional subgroup analyses with respect to the methodology utilized to measure biomarkers in saliva. These included mass spectrometry (MS) versus techniques other than MS such as ELISA and RT‐qPCR (non‐MS). The pooled sensitivity and specificity of salivary biomarkers for BC diagnosis using MS methods were 0.692 (95% CI: 0.675–0.708) and 0.724 (95% CI: 0.708–0.740), respectively (Supplementary file, Figure S11). The PLR, NLR, and DOR of salivary biomarker using MS methods were 2.480 (95% CI: 2.690–2.710), 0.439 (95% CI: 0.403–0.479), and 6.663 (95% CI: 5.578–7.958), respectively (Supplementary file, Figure S11). The AUC for salivary biomarkers detected using MS methods for BC diagnosis was 0.781 (Supplementary file, Figure S12). The sensitivity and specificity of salivary biomarkers for BC diagnosis using non‐MS methods were 0.790 (95% CI: 0.764–0.814) and 0.735 (95% CI: 0.707–0.761), respectively (Supplementary file, Figure S13). The PLR, NLR, and DOR of salivary biomarker using non‐MS methods were 3.000 (95% CI: 2.442–3.685), 0.272 (95% CI: 0.218–0.339), and 12.924 (95% CI: 8.639–19.332), respectively (Supplementary file, Figure S13). The AUC for salivary biomarkers detected using non‐MS methods for diagnosis of BC was 0.859 (Supplementary file, Figure S14). A diagrammatic representation of meta‐regression analysis for the methodology used to measure salivary biomarkers is provided in Figure 8.

#### 3.7.4 Sample size

In the subgroup analysis related to sample size, the pooled sensitivity and specificity, PLR, NLR, and DOR for studies with ≤w55 subjects were 0.773 (95% CI, 0.747–0.798), 0.780 (95% CI, 0.758–0.802), 3.230 (95% CI 2.836–3.678), 0.315 (95% CI, 0.272–0.366), and 13.395 (95% CI, 10.596–16.932), respectively, with the AUC of 0.853 (Supplementary file, Figure S15,S16). The pooled sensitivity, specificity, PLR, NLR, and DOR for studies with >55 subjects were 0.697 (95% CI, 0.681–0.714), 0.701 (95% CI, 0.684–0.718), 2.262 (95% CI, 2.051–2.494), 0.447 (95% CI, 0.405–0.493), and 5.643 (95% CI, 4.649–6.849), respectively, with the AUC of 0.762 (Supplementary file, Figure S17–S19).

#### 3.7.5 Nations

In the Chinese population, the pooled sensitivity and specificity were 0.766 (95% CI: 0.739–0.792) and 0.781 (95% CI: 0.757–0.803). The PLR and NLR were 3.235 (95% CI: 2.827–3.702) and 0.324 (95% CI: 0.279–0.376), respectively, with a DOR of 13.07 (95% CI: 10.320–16.546) and the AUC of 0.851 (Supplementary file, Figure S20–S22). For other nations, the pooled sensitivity was 0.701 (95% CI: 0.684–0.717) and pooled specificity was 0.703 (95% CI: 0.686–0.719). Furthermore, the PLR was 2.292 (95% CI: 2.081–2.525) and the NLR and DOR were 0.439 (95% CI, 0.398–0.485) and 5.888 (95% CI, 4.848–7.150), respectively. The AUC for other nations was 0.767 (Supplementary file, Figure S23–S25). HSROC curves for subgroup analyses based on saliva type (stimulated & unstimulated), biomarker type (proteomics and metabolomics), and countries are illustrated in Figure S26–S28 of the Supplementary file, respectively.

**FIGURE 9:**
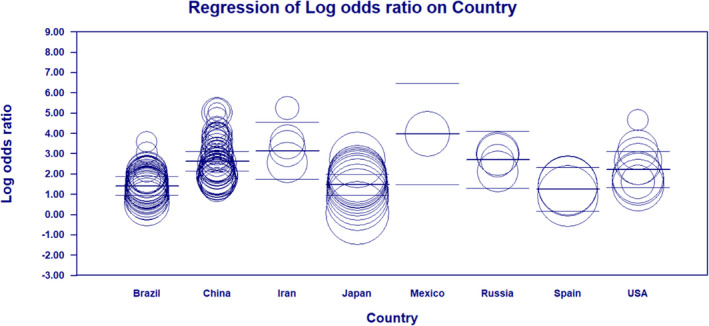
Scatter plot of meta‐regression based on the country where the study was conducted

Figure 9 provides a diagrammatic representation of meta‐regression analysis based on the country. Figure 9 shows that irrespective of the number and diameter of circles representing the number of studies and the sample size, studies with progressively higher DOR were conducted in Mexico, Iran, and China, respectively.

### 3.8 Quality assessment enhanced SROC plot

The results of assessment for bias and applicability in the analyzed studies using the glyph system. Are depicted in Figures S29 and S30 of the Supplementary file. These graphs provide a quick visual overview of the quality of eligible studies and help to identify which ones are at high‐bias risk or applicability concerns. They also allow for comparison between the number of high‐ and low‐(or uncertain) risk studies. For example, as seen in Figure S30C of the Supplementary file, most studies with high‐bias risk in relation to patient selection are above the SROC line, while in Figure S30B of the Supplementary file, most studies with high‐bias risk concerning index test are below the SROC line. Figure S31 of the Supplementary file provides a visual representation of the total risk of bias and applicability concerns for the studies included in our meta‐analysis.

### 3.9 Meta‐regression

Meta‐analysis showed general heterogeneity. To determine the sources of heterogeneity between studies, meta‐regression using the following covariates as an independent predictor variable was performed; Saliva type (stimulated and unstimulated), type of biomarker (proteomics, metabolomics), sample size >55 and ≤55, and the mean age of patients >52 and ≤52 (Table 3).

**TABLE 3:** Meta‐regression of covariates as an independent predictor variable for BC diagnosis using salivary biomarkers

Covariate	Coefficient	Standard error	95% CI	Z‐value	*p* (two‐sided)
Intercept	0.6692	1.4531	−2.1788, 3.5172	0.46	0.6451
Mean age of patients	0.0321	0.0321	−0.0308, 0.0949	1	0.3176
Sample size	−0.0092	0.0022	−0.0135, −0.005	−4.25	0.0001
Type of biomarker	−0.0492	0.093	−0.2315, 0.1332	−0.53	0.5971
Saliva type	0.7929	0.2568	0.2896, 1.2963	3.09	0.002

*Note*: Q = 43.83, df = 4, *p* = 0.0000.

*Note*: τ^2^ = 0.2559, τ = 0.5059, I^2^ = 42.16%, Q = 200.57, df = 116, *p* = 0.0000.

### 3.10 Publication bias of Meta‐analysis

**FIGURE 10:**
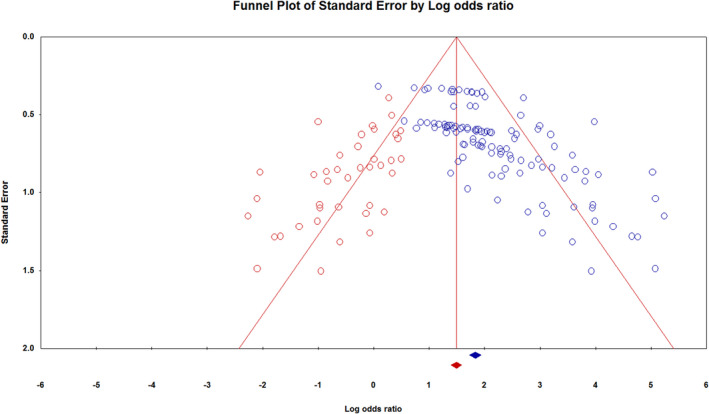
Funnel plot of observed and imputed studies. The blue circles represent the observed study units, and the red circles represent studies trimmed on the left side

Comprehensive Meta‐Analysis (CMA) v.3.3.070 (Biostat, USA) was applied to perform Egger's statistical tests. The analysis showed that Egger's model intercept (B0) was 2.769 (95% CI: 2.221–3.372), t‐value was 9.621, and df was 119. The p‐value for both one‐tailed and two‐tailed analysis was <0.001. Using Duval and Tweedie, the adjusted value of point estimate and Q‐value were 4.677 and 558.486, respectively (Figure 10).

## 4 DISCUSSION

Our systematic review was based on 14 eligible studies with 121 study units, including 4149 cancer patients, 4136 healthy control participants, and 354 control subjects with benign breast disease. Our analysis showed that, based on Mandrekar criterion,^59^ salivary biomarkers had “excellent” diagnostic accuracy for BC. When we applied the Jones and Athanasiou criterion,^60^ our analysis indicated that salivary markers have “good” diagnostic accuracy for BC with the sensitivity of 0.72, specificity of 0.73, and the AUC of 0.802. In our analysis, pooled PLR value of 2.58 indicated that BC patients were 2.58 times more likely to have a positive test result compared to the control group without cancer. In addition, the pooled NLR value of 0.39 indicated that the possibility of BC diagnosis despite a negative salivary diagnostic test was 39%. The pooled DOR of 7.9 in our analysis shows an overall moderate diagnostic accuracy for salivary biomarkers in the diagnosis of BC and “very good” diagnostic accuracy based on the AUC value.^60^, ^61^ Fagan's nomogram comprehensively considers PLR and NLR and adjusts the likelihood ratios according to the prior probability of diagnosis for salivary biomarkers. Fagan's nomogram results showed that salivary biomarkers could be helpful for clinical decision‐making for BC detection.

The meta‐regression analysis showed that increasing the mean age of patients increased the DOR. The increase in DOR for salivary biomarkers with increasing age could be related to the higher possibility of BC in older individuals^62^ and the possibility of changes in the saliva composition with aging.^63^ This could imply an increase in the diagnostic power of saliva with increasing age. Therefore, it is warranted that future studies investigate the impact of age on the accuracy of salivary diagnostics for BC.

Our results show unstimulated saliva has a more acceptable diagnostic value than stimulated saliva (Supplementary file, Figure S26). Similar results have been reported by Ventura et al., who compared the protein levels in the stimulated and unstimulated saliva and found that the latter seems to have a better diagnostic value.^64^ Similar observations have also been reported for stimulated and unstimulated saliva with respect to the microbiome and chemical analysis.^65^, ^66^

As summarized in Table S2, the highest DOR with respect to the methodology was 152.50 for RT‐qPCR technique, 53.330 for SERS methods, 20.044 for western blot, and 12.754 for ELISA, respectively. The lowest DOR of 3.486 was related to the Immunoassays method. Our findings indicate that RT‐qPCR, SERS, and western blot, respectively, have the highest DOR for salivary diagnosis of BC. In addition, compared to MS methods with pooled DOR of 6.663, ELISA had a higher DOR of 12.754 for the salivary diagnosis of BC.

Meta‐regression analysis showed that variation in the sample size and type of saliva type collected contributed to the heterogeneity (*p* < 0.05). Although the Cochrane handbook^67^ does not cite evidence of considerable heterogeneity for the systematic review of interventions, variation in the sample size (*p* = 0.0001), and saliva type (*p* = 0.002) were the main contributors to the heterogeneity in our systematic review (Table 3 and Table S2 of Supplementary file).

Comparison of AUC and DOR values of HSROC based on the biomarker type showed that salivary proteomics are better than salivary metabolomics in discriminating between patients with and without BC (Supplementary file, Figure S27). Although the role of salivary metabolite biomarkers as cancer markers has been studied in previous studies,^68^, ^69^, ^70^ in accordance with our results, Rapado‐González et al. reported higher sensitivity and specificity for proteomics than metabolomics in cancer diagnosis.^40^

Our comparison of the results of studies in the Chinese population with investigations conducted in other populations showed that, regardless of the methodology, salivary biomarkers can be used efficiently and effectively to diagnose BC in Chinese patients. Some studies have reported that biomarker accuracy in the Chinese population is different from other countries.^71^, ^72^

Although a number of systematic reviews have examined the utility of saliva in the diagnosis of oral, head, and neck malignancies,^73^, ^74^, ^75^, ^76^, ^77^, ^78^, ^79^ limited review studies on the use of salivary biomarkers for diagnosis of distant cancers, especially BC are available.^54^ To the best of our knowledge, this is the first systematic review and meta‐analysis evaluating the potential of salivary biomarkers in the diagnosis of BC. Rapado‐González and co‐workers previously evaluated the application of salivary diagnostics in pancreatic, esophageal, gastric, lung, ovarian, and BC. They claimed that saliva is a promising noninvasive source of biomarkers for distant malignant non‐oral tumors, with an accuracy of 85%. However, the implications of mean age of patients, saliva type, biomarker type, biomarker measurement method, sample size, and nations were not examined in relation to the malignancies. In their review, Porto‐Mascarenhas and co‐workers concluded that salivary biomarkers are more reliable for the diagnosis of advanced stages compared to early stages of BC^54^ and suggested examination of saliva metabolites for BC diagnosis. While adherent to the search strategy and following inclusion and exclusion criteria, all review studies are subject to the risk of bias. We have reported the effects of mean age of patients, saliva type, biomarker measurement method, sample size, biomarker type, and nations on the diagnostic value of saliva using meta‐analysis. Collectively, these findings suggest that saliva‐based diagnostic tests may be a promising tool for BC screening because biomarkers detected in saliva can distinguish BC patients from healthy individuals with high accuracy; however, there were a number of limitations to our analysis.

One of the main limitations is dissemination bias because studies with positive diagnostic results are more accessible than those which reveal negative findings.^80^, ^81^ As seen in Figure 10, which illustrates the publication bias of our analysis, the trimmed studies are represented as red circles to the left of the funnel plot for observed and imputed studies. Another drawback is the risk of bias in small, unmatched studies, which cannot be controlled. Another limitation of this meta‐analysis that could affect our results was the inclusion of studies with small sample size which could lead to higher bias risk. In addition, our analysis was potentially subject to a variety of confounders because many of the articles reviewed did not provide adequate information. For example, the majority of studies did not specify the type of BC. Moreover, only a limited number of studies provided information about tumor, node, metastasis (TNM) stage, or tobacco and alcohol use history. Many studies did not report correlation analysis between TNM stages and salivary biomarkers either. Therefore, controlled studies with larger sample sizes with clinical and demographical details of BC and matching are needed to confirm and provide additional evidence for clinical application of salivary biomarkers in BC diagnosis. The current study also has a number of strengths. We used systematic review and meta‐analysis to unravel the value of salivary biomarkers for the BC diagnosis, a malignancy distant from the oral cavity. We also utilized a graphical quality assessment enhanced SROC plot to display the results of each study with multiple indicators using QUADAS‐2. Our analysis showed that unstimulated saliva could have higher diagnostic accuracy for BC. The results of this study suggest that combinations of transcriptomic and proteomic data, as well as clinical information help to improve future studies and potential applications for clinical diagnosis.

## 5 CONCLUSION

Our meta‐analysis showed that saliva might be a promising, efficient, and noninvasive biomarker with the potential to differentiate between patients with and without BC. Future large‐scale studies that appropriately account for various potential confounders such as age, race, tobacco, and alcohol consumption, type of BC disease stage, biomarker type, methodology, and defined threshold values could confirm and fine‐tune the efficacy of salivary biomarkers before clinical implementation in BC detection. This is the first systematic review with meta‐analysis, ‐regression, and graphical quality assessment to investigate the potential of salivary biomarkers for BC diagnosis. Despite the limitations, salivary biomarkers offer the promise of sufficient sensitivity and specificity in the diagnosis of BC, but randomized validation steps are recommended before clinical applicability.

## CONFLICT OF INTEREST

The authors declare no potential conflict of interest.

## AUTHOR CONTRIBUTIONS

MK, MF, and SK have contributed to the conception and design of the work. MK and SK independently evaluated the literature. MK, SK, and SM reviewed the articles based on the inclusion criteria by reviewing the titles, abstracts, and full texts of each study. MK and SK contributed to the acquisition and analysis of the data. MK and MF drafted the manuscript. MF and SK revised the manuscript. All authors have read and approved the final version of the submission.

## Supporting information


Figure S1–S31

**Table S1** Risk of bias assessment and applicability concerns using QUADAS‐2 of included studies.

**Table S2** Subgroup analysis of saliva for breast cancer diagnosis based on different covariates.

## Data Availability

The datasets used and analyzed during the current study are available from the corresponding author upon reasonable request.
