# Supplementary material for: Salivary biomarkers in breast cancer diagnosis: A systematic review and diagnostic meta‐analysis
Source: Cancer Med. 2022 Mar 22;11(13):2644–61. doi: 10.1002/cam4.4640 (PMC9249990; doi:10.1002/cam4.4640)
Supplement: Supplementary file 1 — Figure S1–S31 [file CAM4-11-2644-s003.docx]

Supplementary Materials: Salivary biomarkers in breast cancer diagnosis: A systematic review and diagnostic meta-analysis

Maryam Koopaie, Sajad Kolahdooz, Mahnaz Fatahzadeh, Soheila Manifar


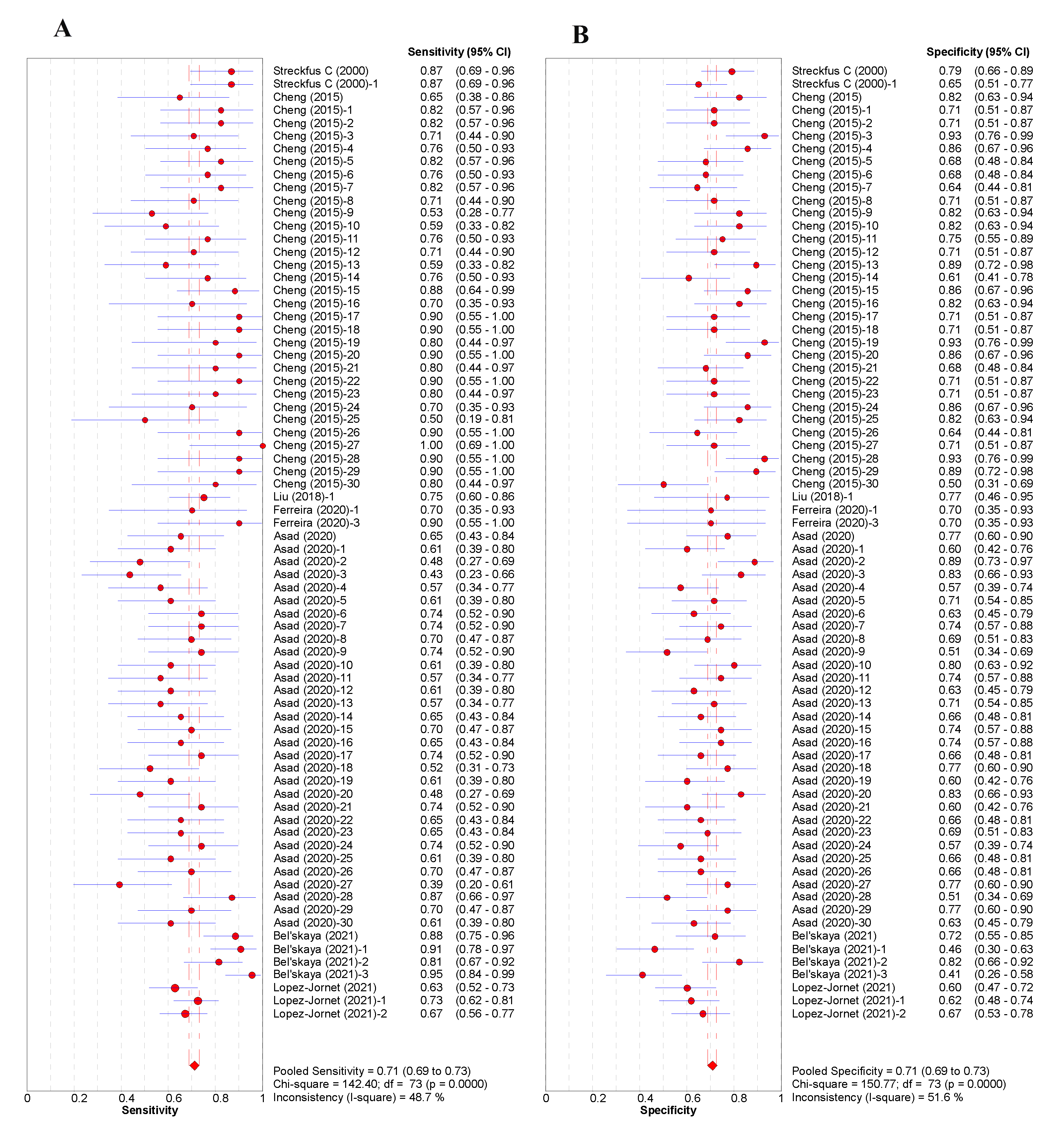


Figure S1. Forest plots of A) sensitivity and B) specificity for salivary biomarkers in diagnosis of breast cancer in patients with the mean age of equal or less than 52 years old (95% CI).


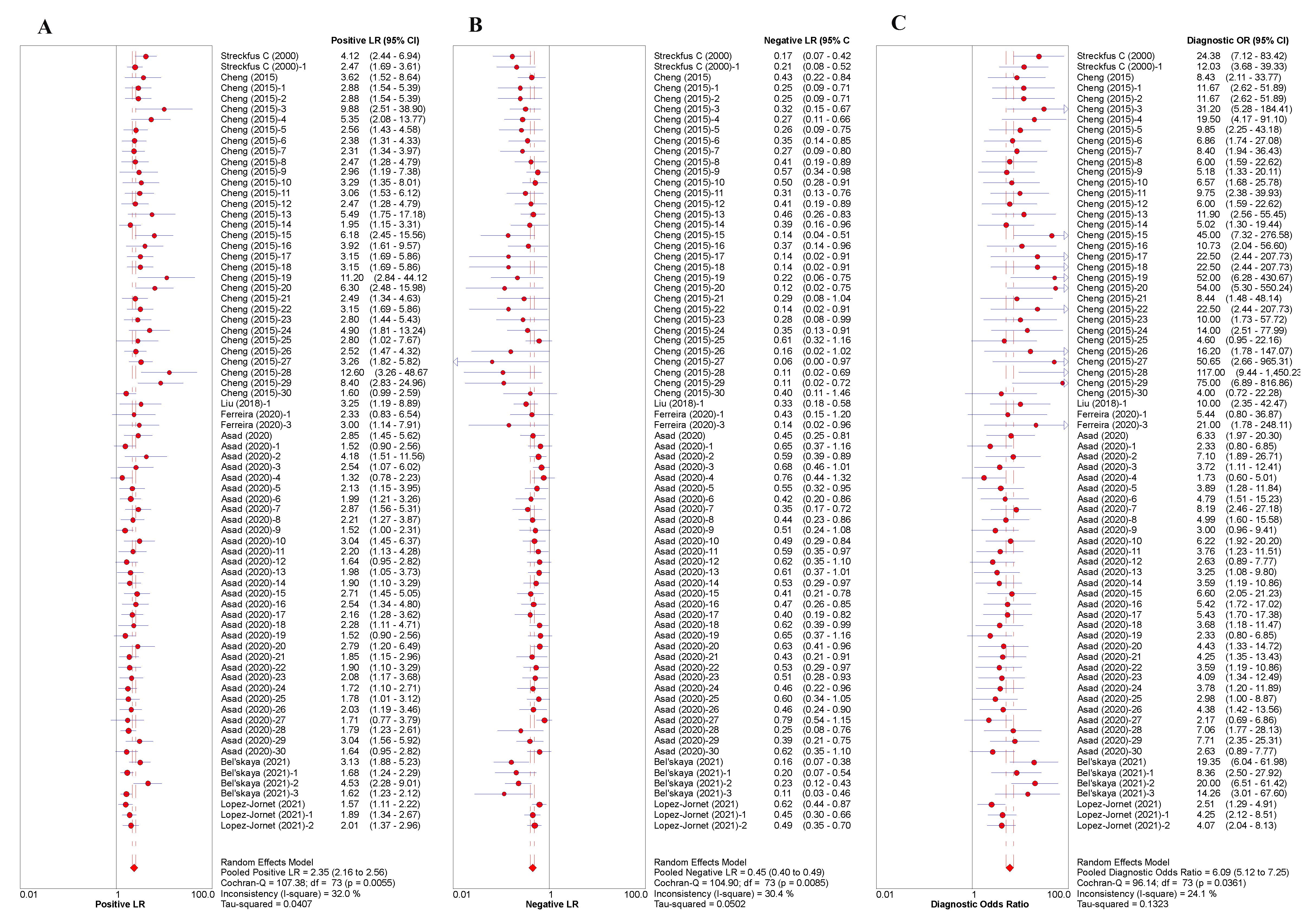


Figure S2. Forest plots of A) PLR, B) NLR, and C) DOR for salivary biomarkers in diagnosis of breast cancer in patients with the mean age of equal or less than 52 years old (95% CI).


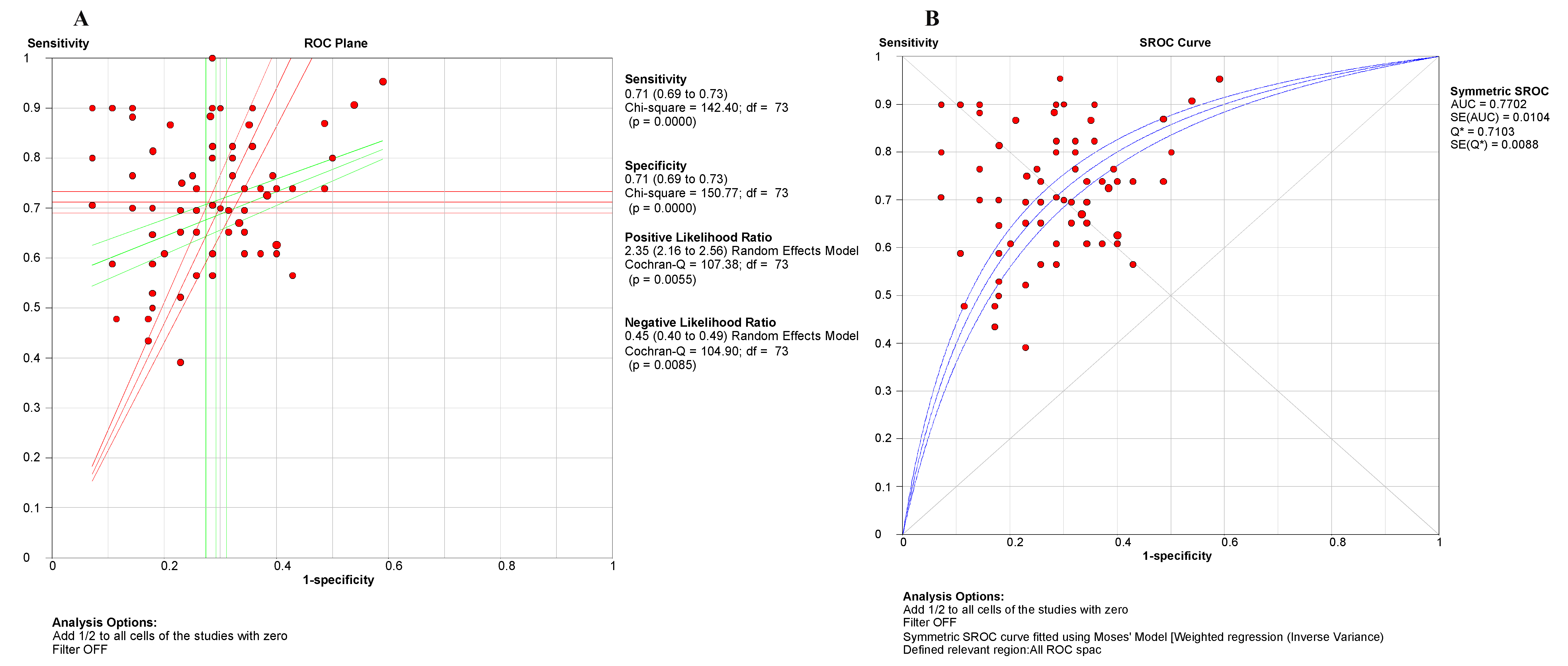


Figure S3. A) ROC plane and B) SROC curve for salivary biomarkers in diagnosis of breast cancer in patients with the mean age of equal or less than 52 years old.


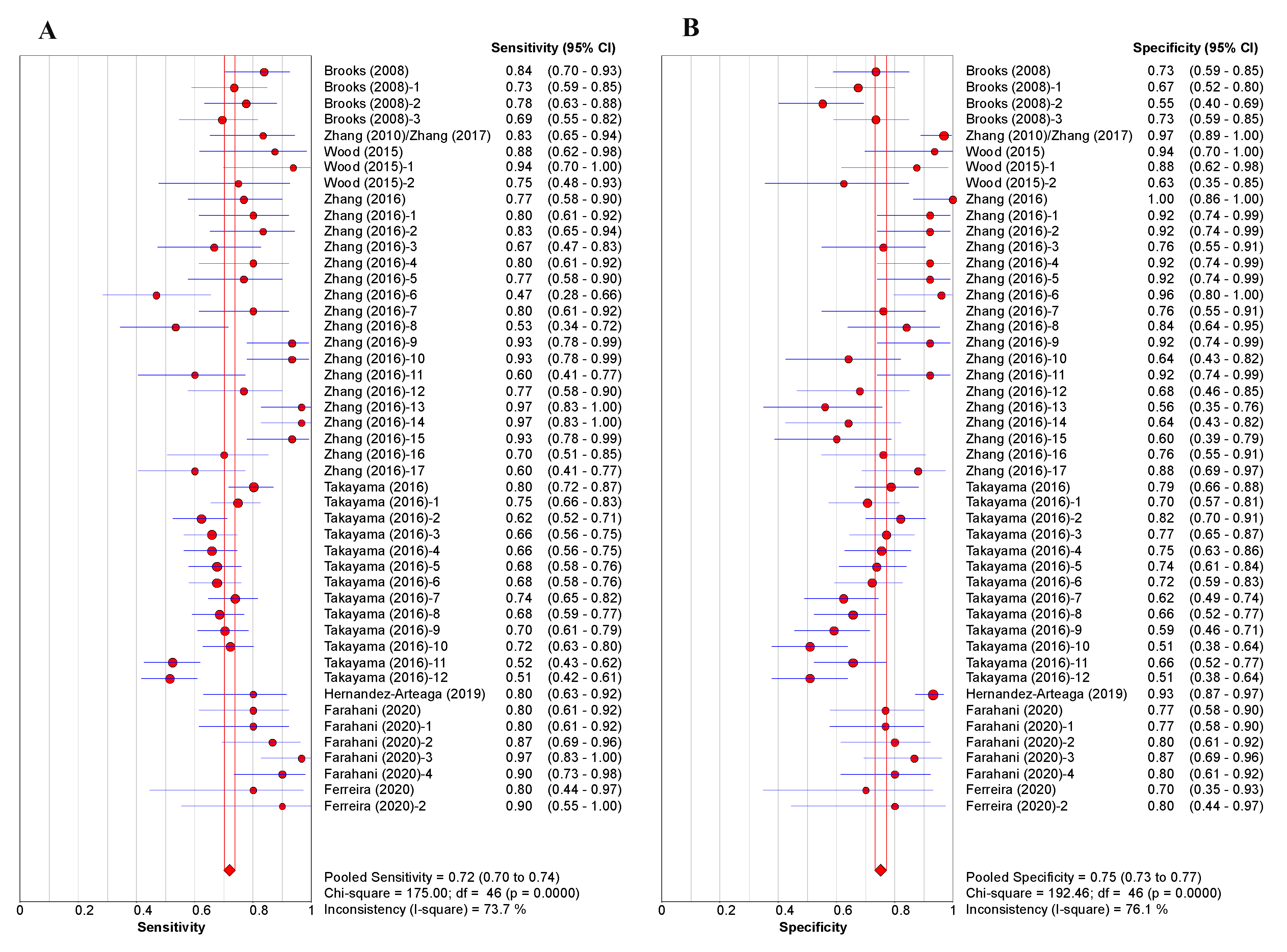


Figure S4. Forest plots of A) sensitivity and B) specificity for salivary biomarkers in diagnosis of breast cancer in patients with the mean age of more than 52 years old (95% CI).


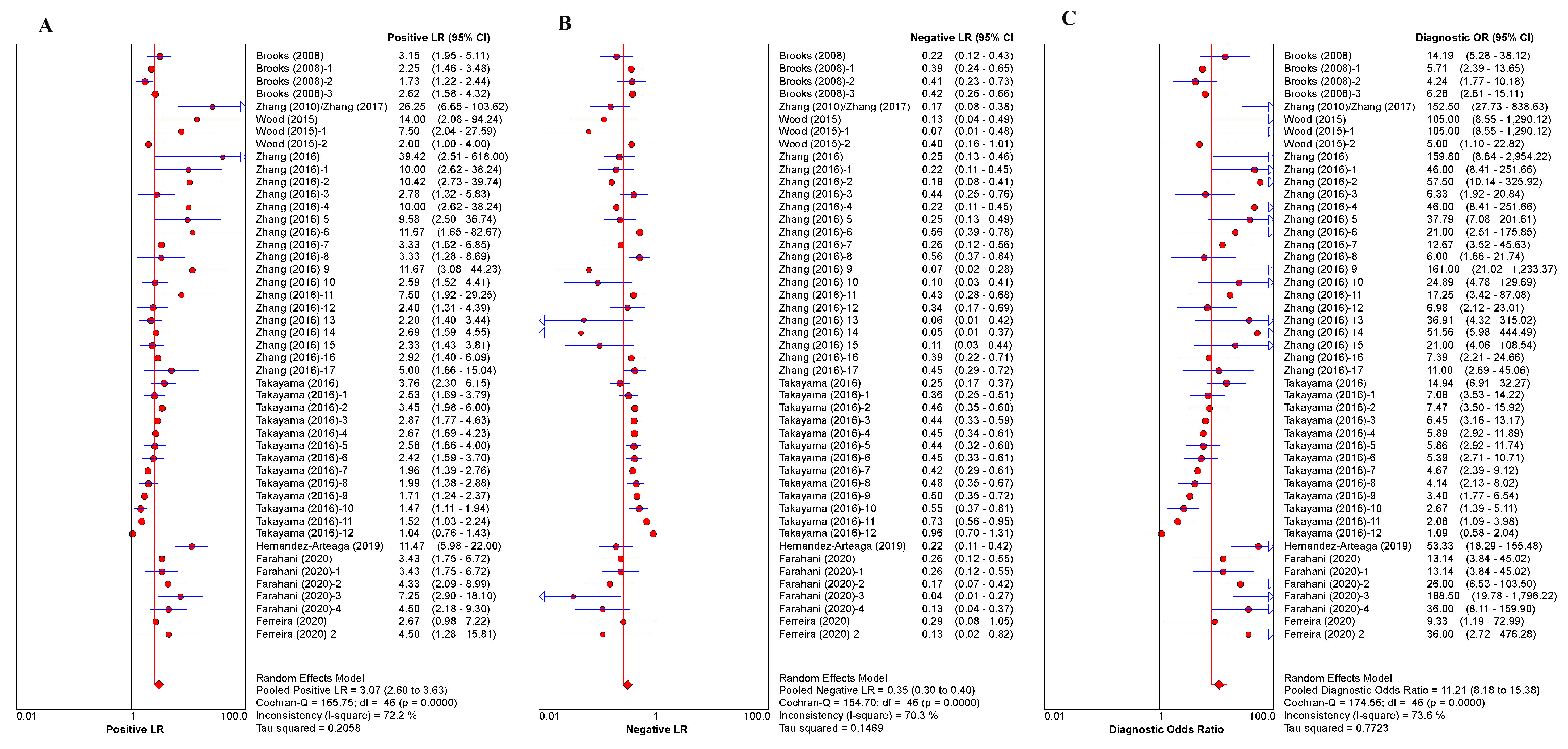


Figure S5. Forest plots of A) PLR, B) NLR, and C) DOR for salivary biomarkers in patients with the mean age of more than 52 years old (95% CI).


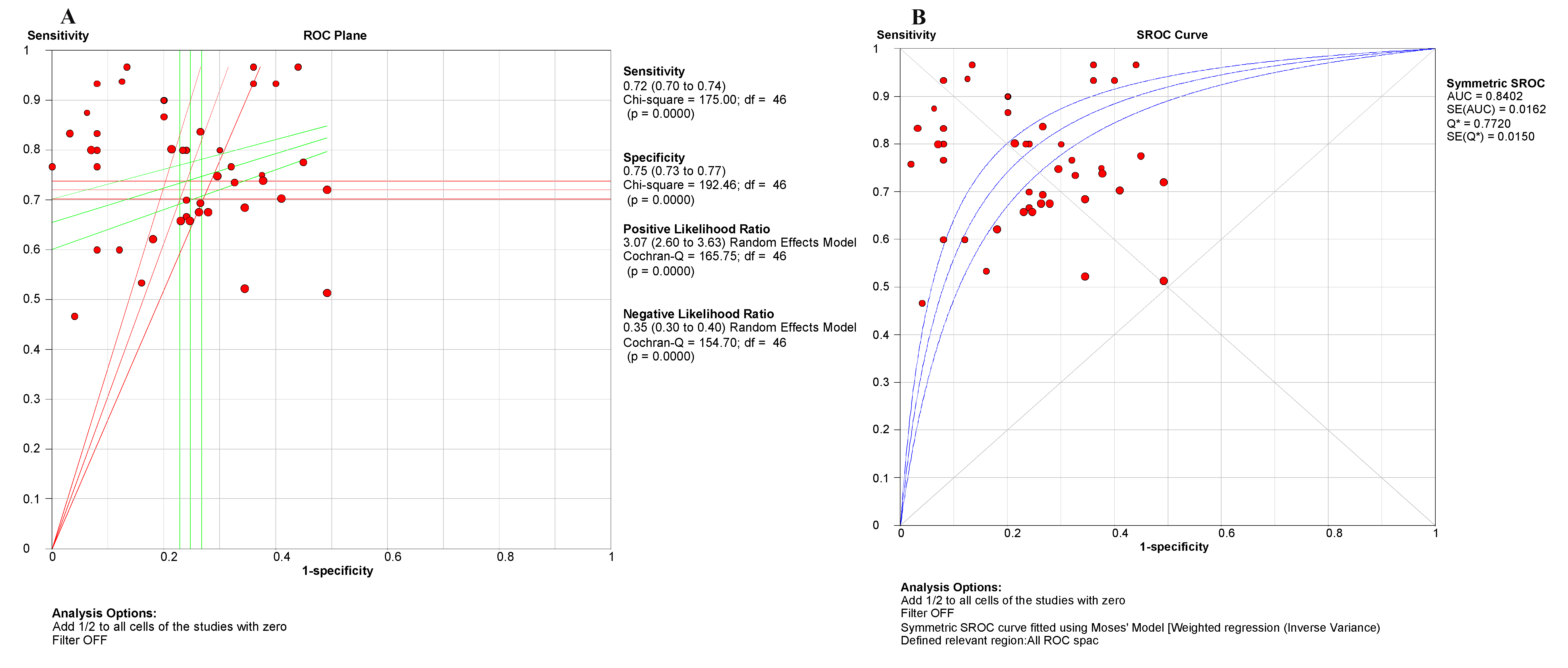


Figure S6. A) ROC plane and B) SROC curve for salivary biomarkers in diagnosis of breast cancer in patients with the mean age of more than 52 years old.


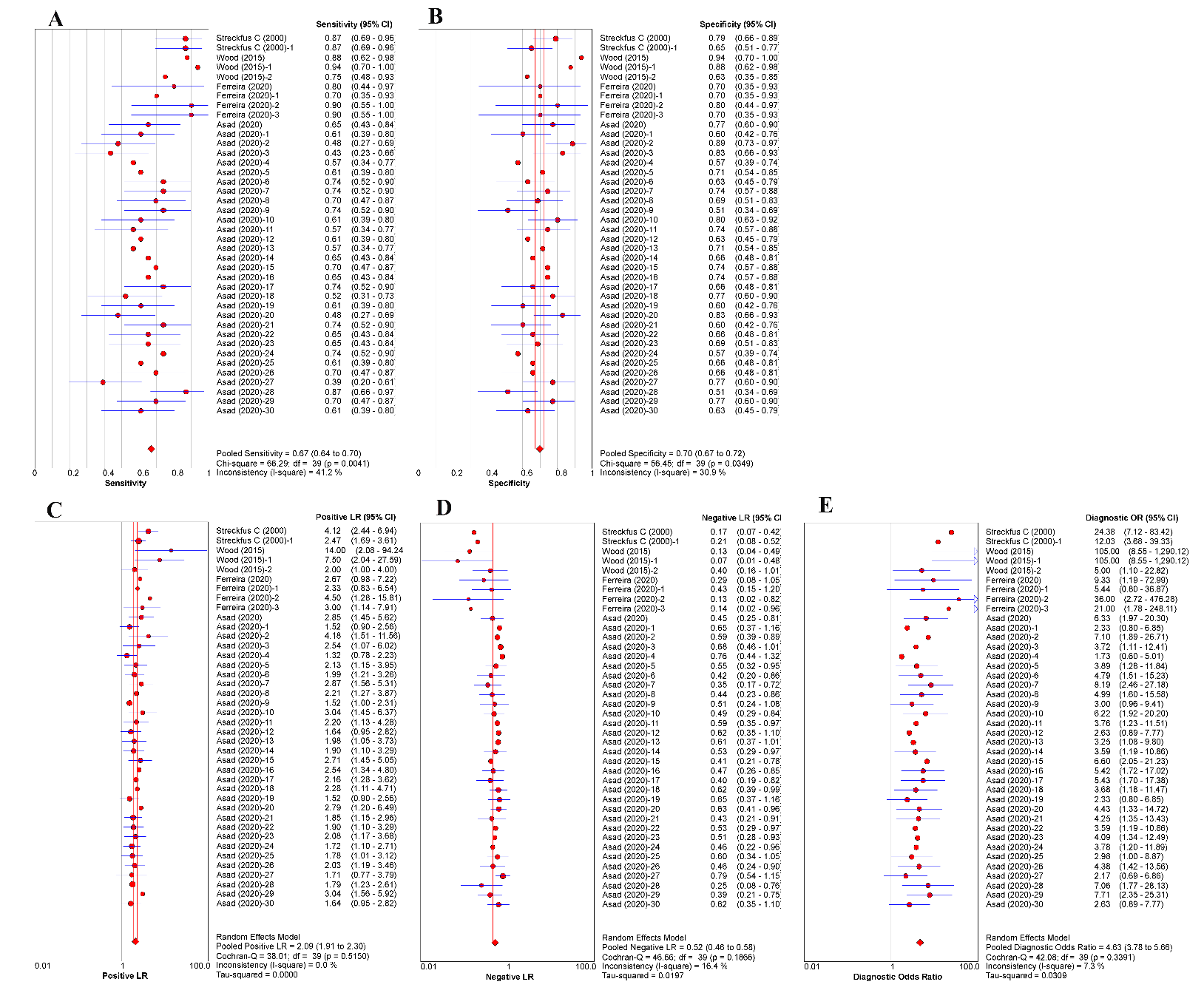


Figure S7. Forest plots of A) sensitivity, B) specificity, C) PLR, D) NLR, and E) DOR for stimulated salivary biomarkers in the diagnosis of breast cancer (95% CI).


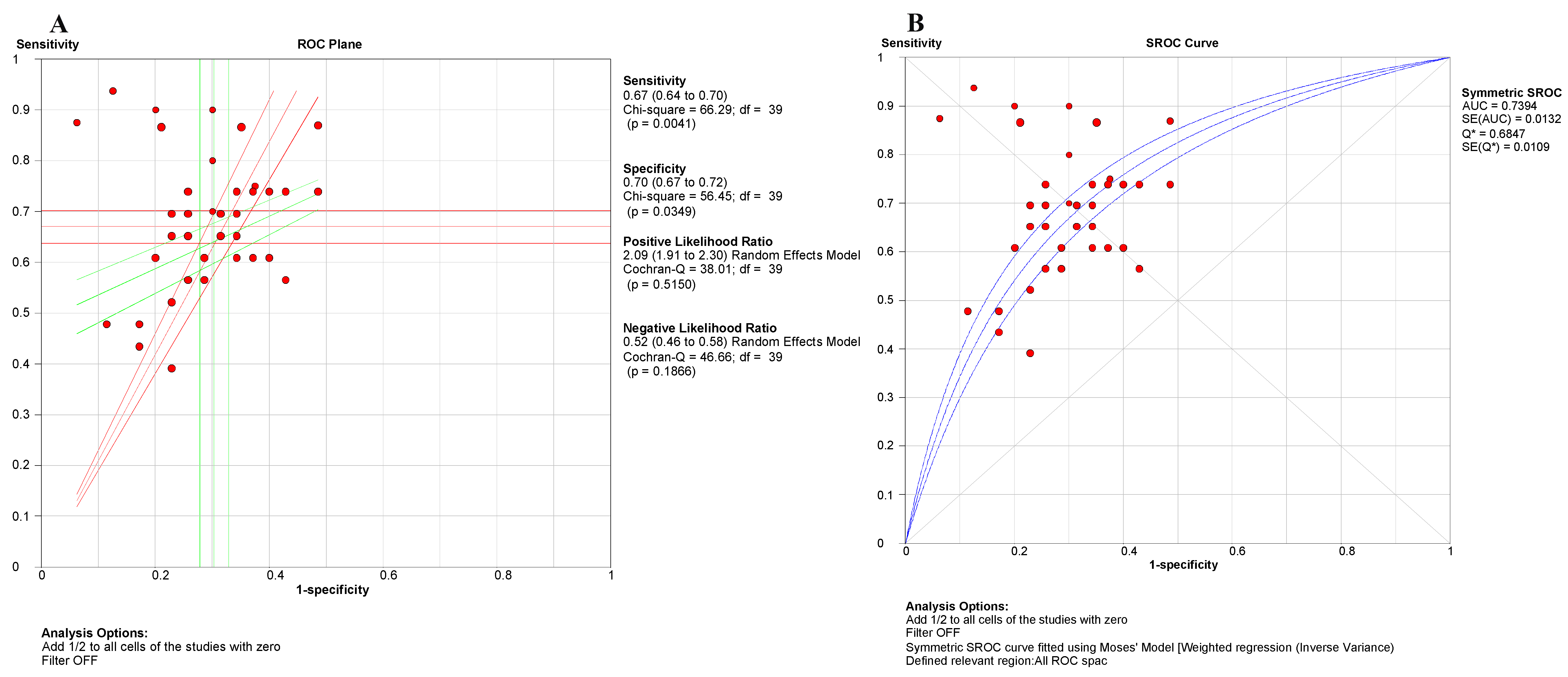


Figure S8. A) ROC plane and B) SROC curve for stimulated salivary biomarkers in diagnosis of breast cancer.


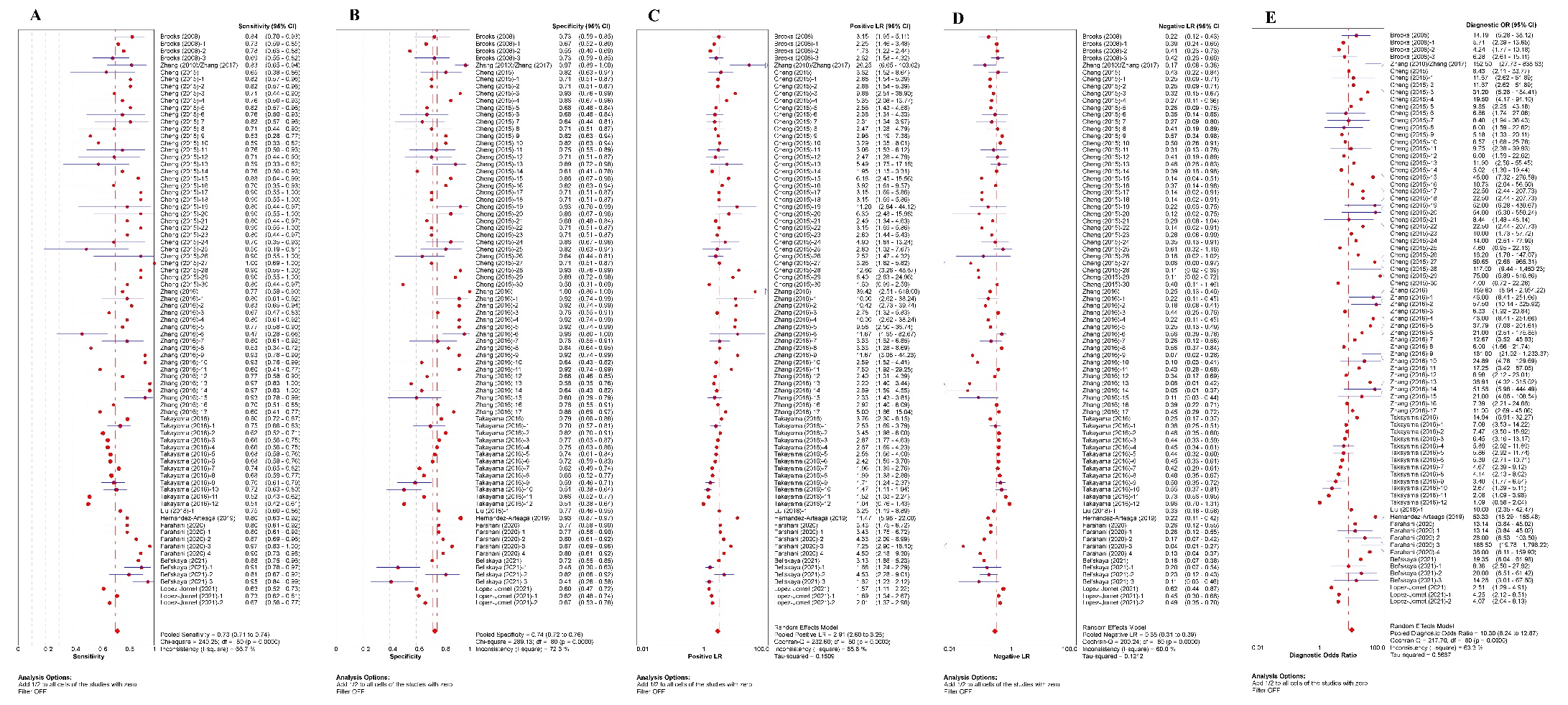


Figure S9. Forest plots of A) sensitivity, B) specificity, C) PLR, D) NLR, and E) DOR for unstimulated salivary biomarkers in diagnosis of breast cancer (95% CI).


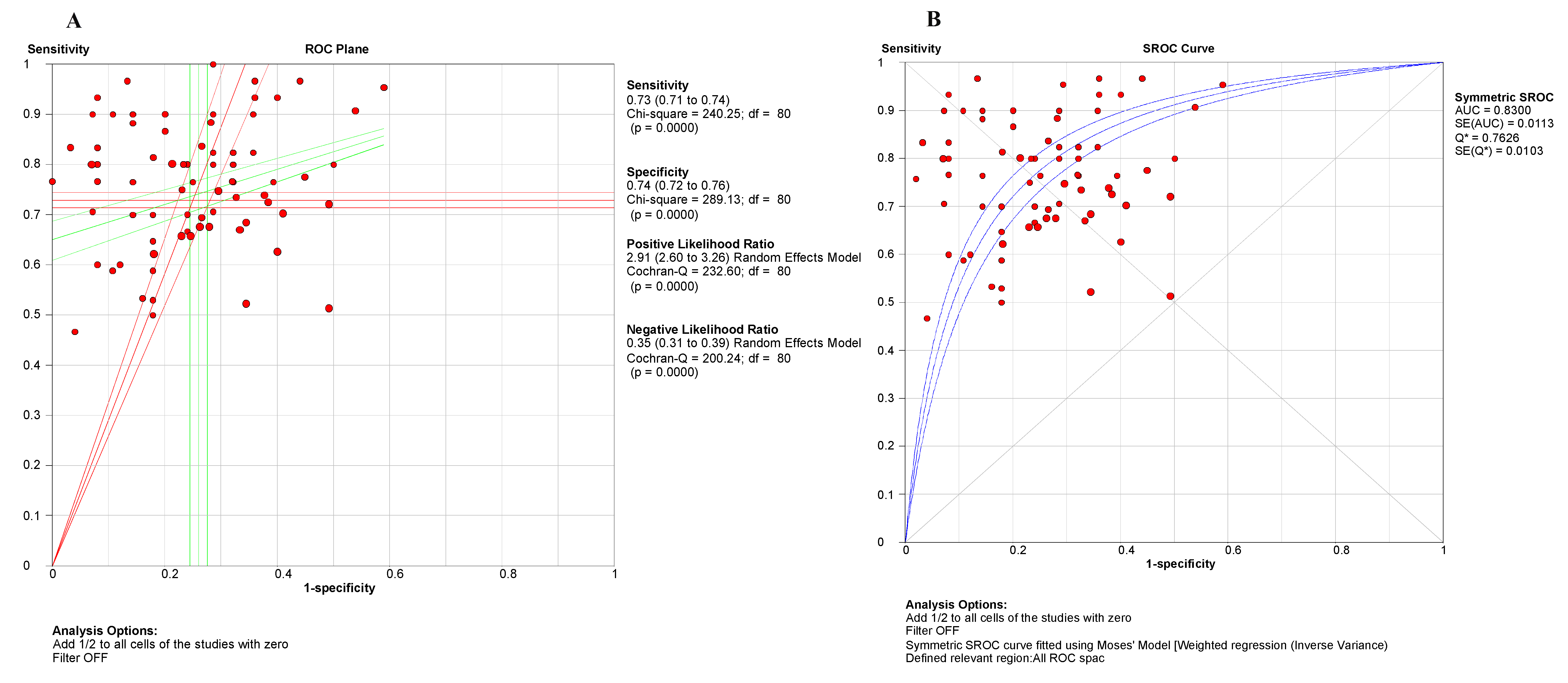


Figure S10. A) ROC plane and B) SROC curve for unstimulated salivary biomarkers in diagnosis of breast cancer.


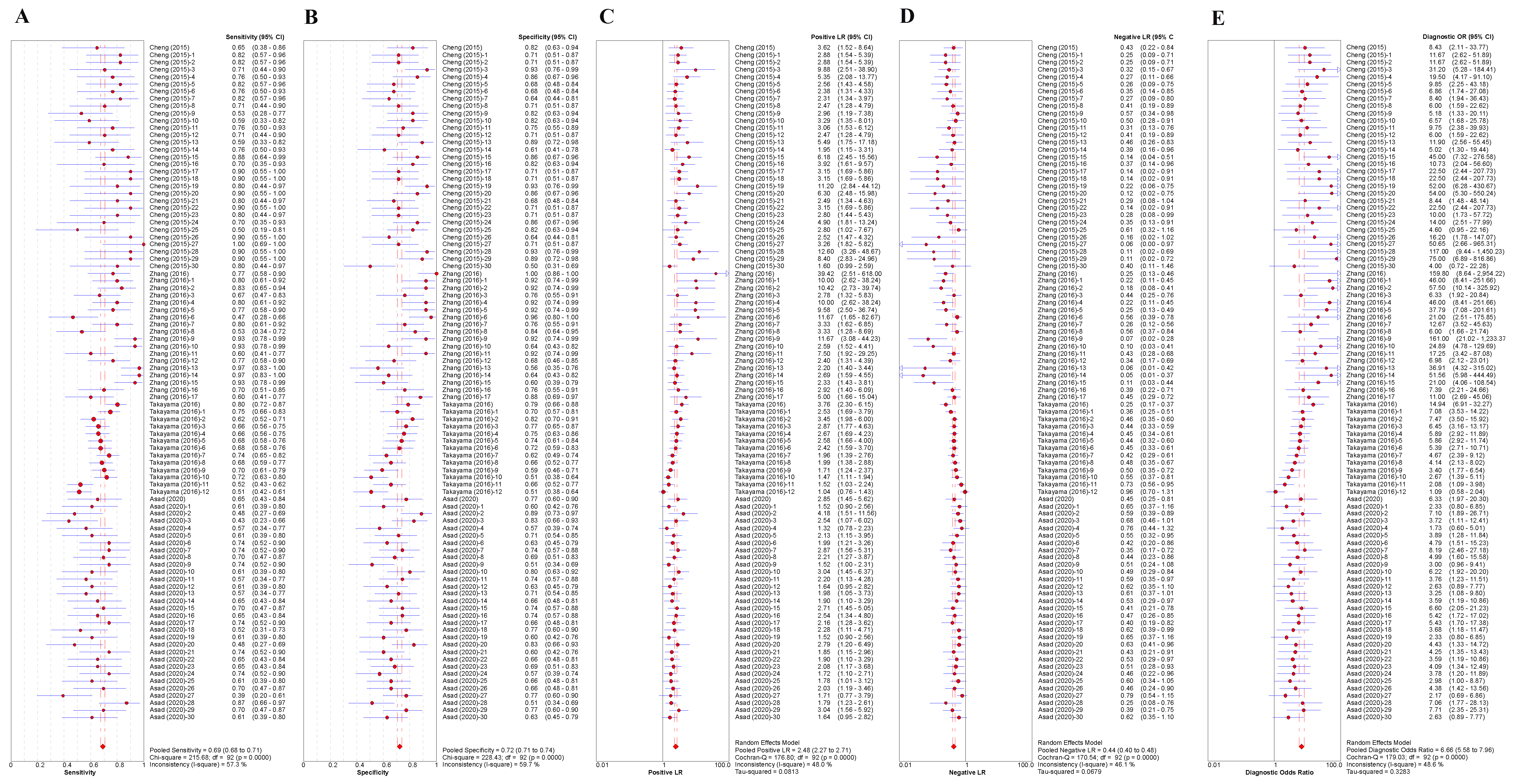


Figure S11. Forest plots of A) sensitivity, B) specificity, C) PLR, D) NLR, and E) DOR for mass spectrometry (MS) measurement methods of salivary biomarkers in diagnosis of breast cancer (95% CI).


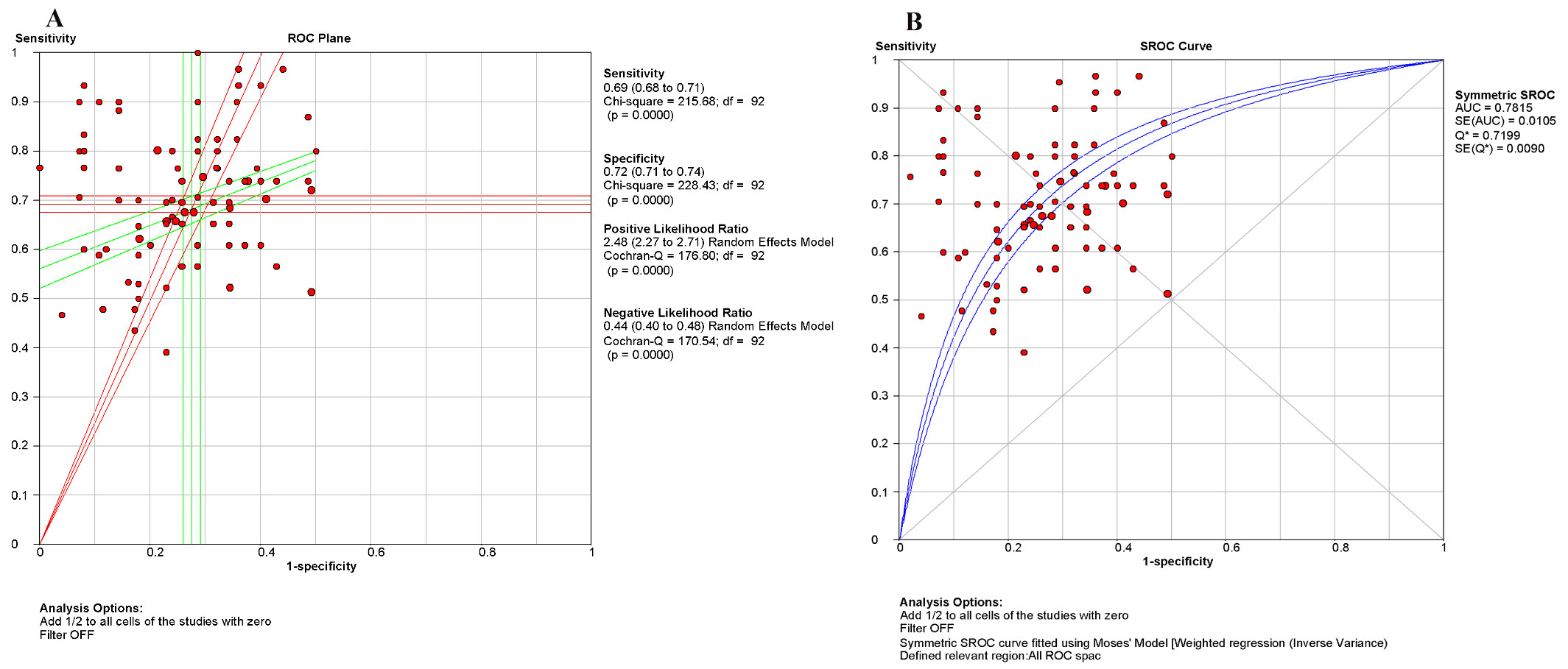


Figure S12. A) ROC Plane and B) SROC curve for mass spectrometry (MS) measurement methods of salivary biomarkers in diagnosis of breast cancer.


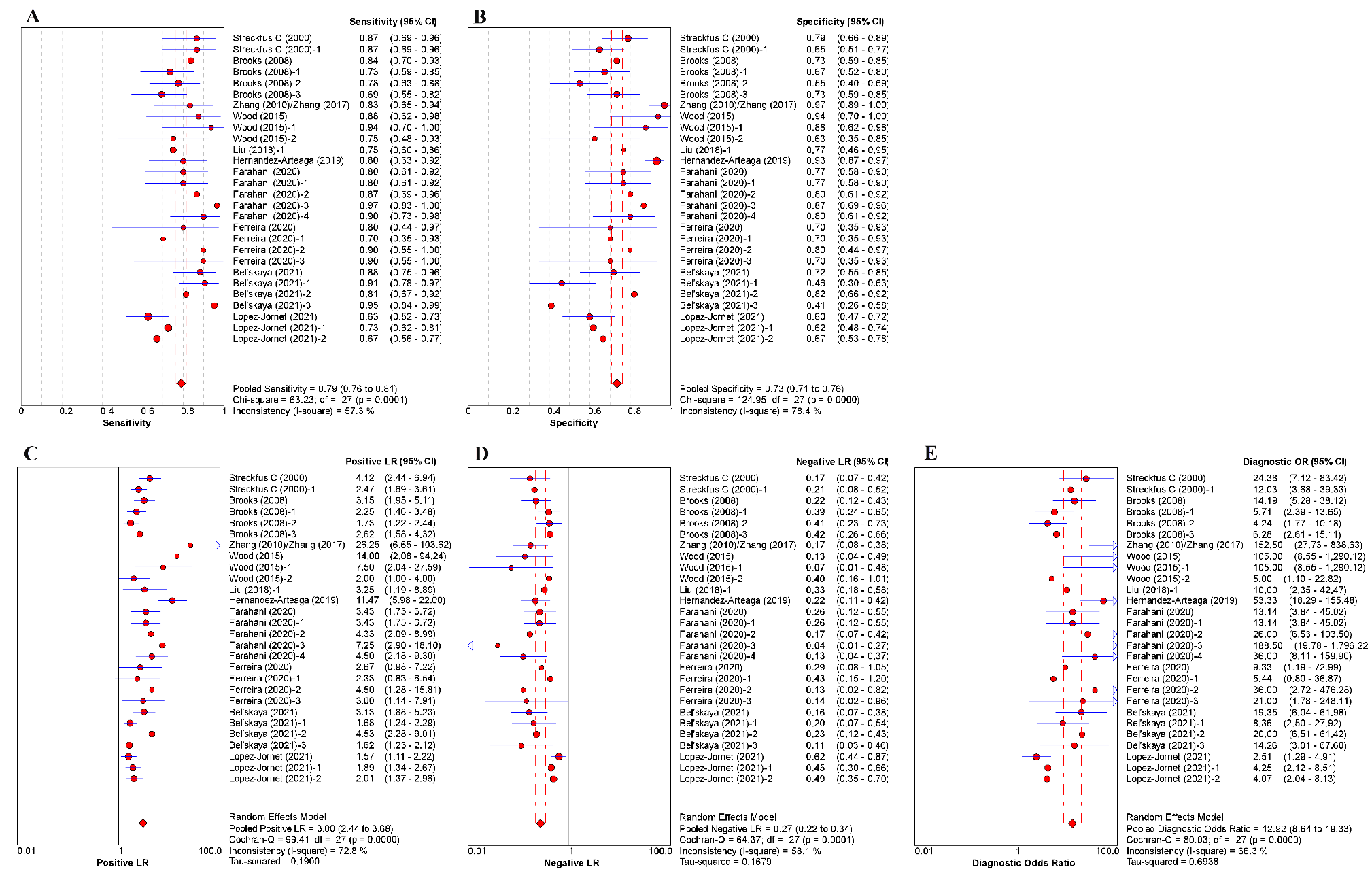


Figure S13. Forest plots of A) sensitivity, B) specificity, C) PLR, D) NLR, and E) DOR for non-mass spectrometry (non-MS) measurement methods for salivary biomarkers in diagnosis of breast cancer (95% CI).


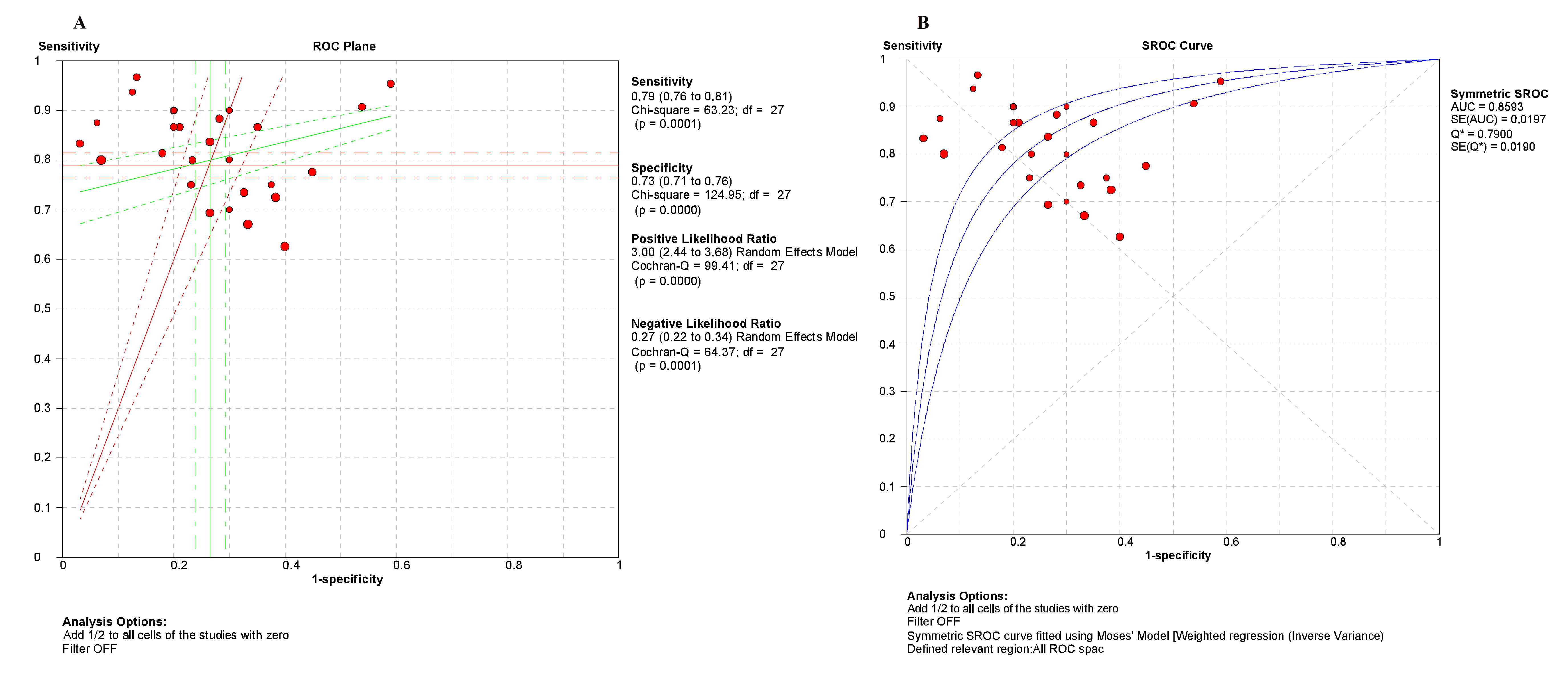


Figure S14. A) ROC plane and B) SROC curve for non-mass spectrometry (non-MS) measurement methods for salivary biomarkers in diagnosis of breast cancer.


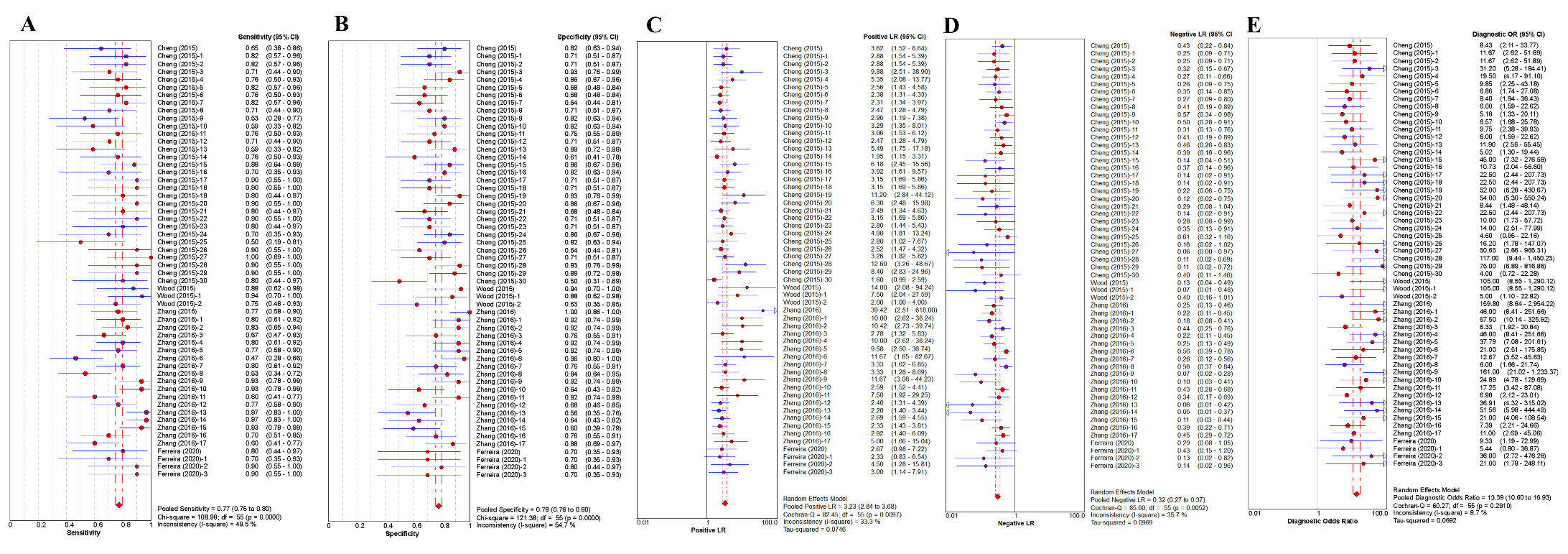
Figure S15. Forest plots of A) sensitivity, B) specificity, C) PLR, D) NLR, and E) DOR for sample size equal or less than 55 subjects in diagnosis of breast cancer using salivary biomarkers (95% CI).


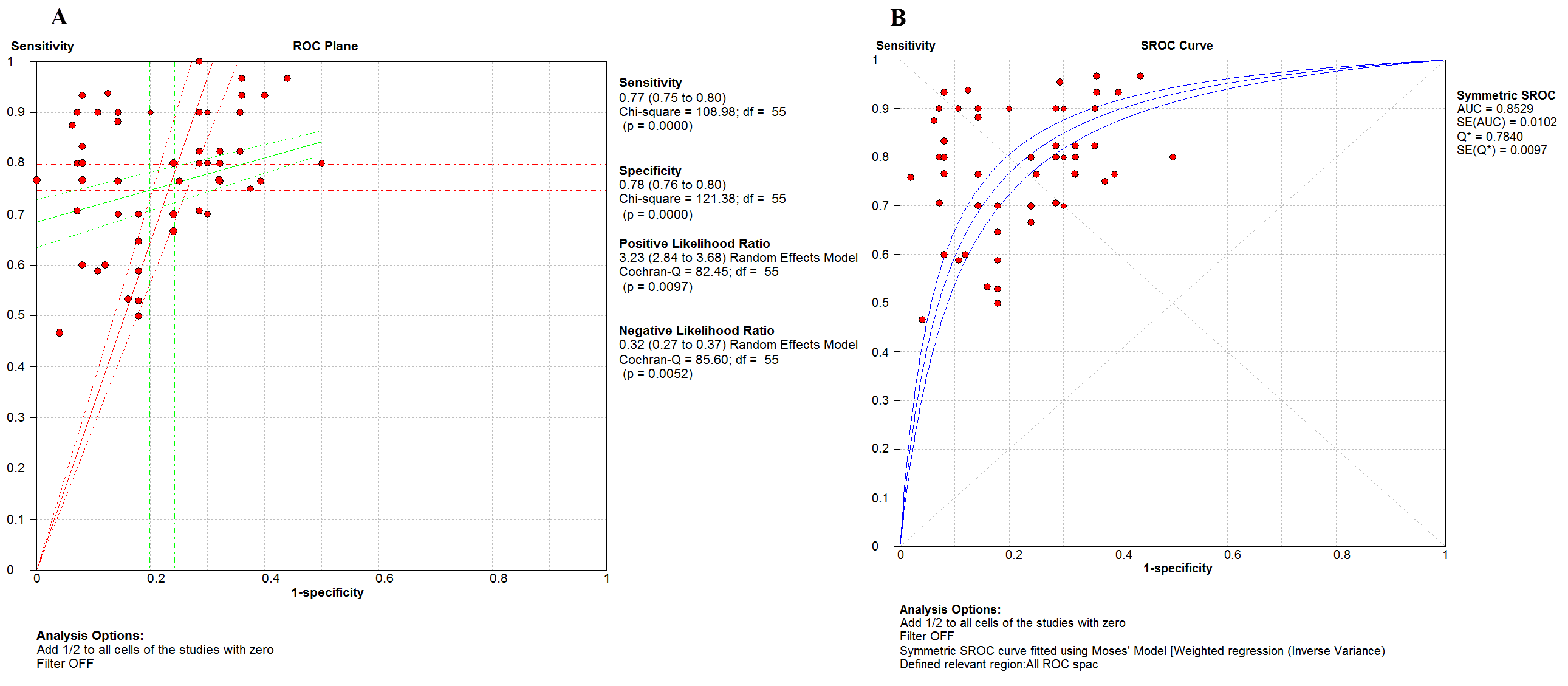


Figure S16. A) ROC plane and B) SROC curve for sample size equal or less than 55 subjects in diagnosis of breast cancer using salivary biomarkers.


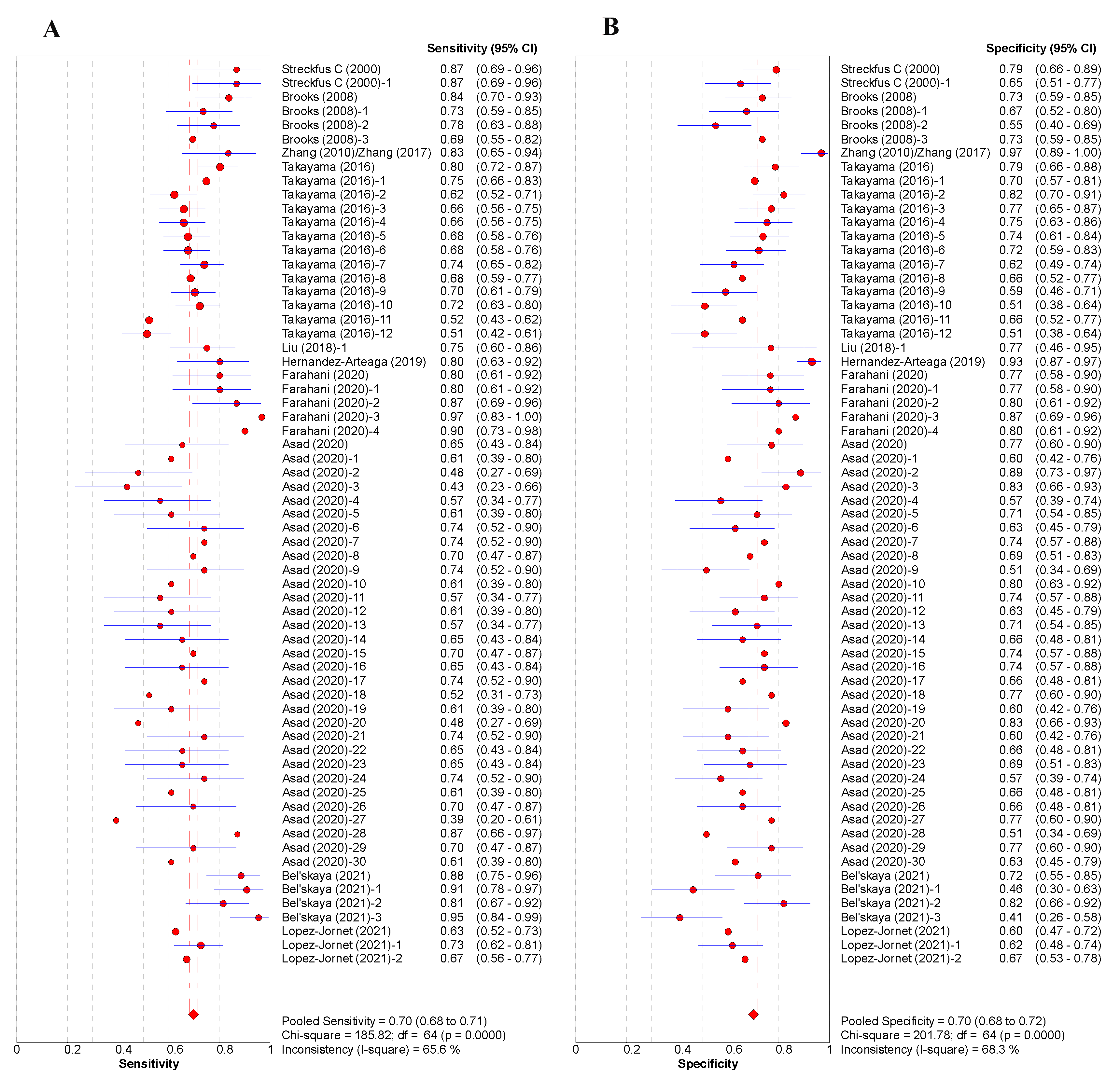


Figure S17. Forest plots of A) sensitivity, B) specificity, C) PLR, D) NLR and E) DOR for sample size more than 55 subjects in diagnosis of breast cancer using salivary biomarkers (95% CI).


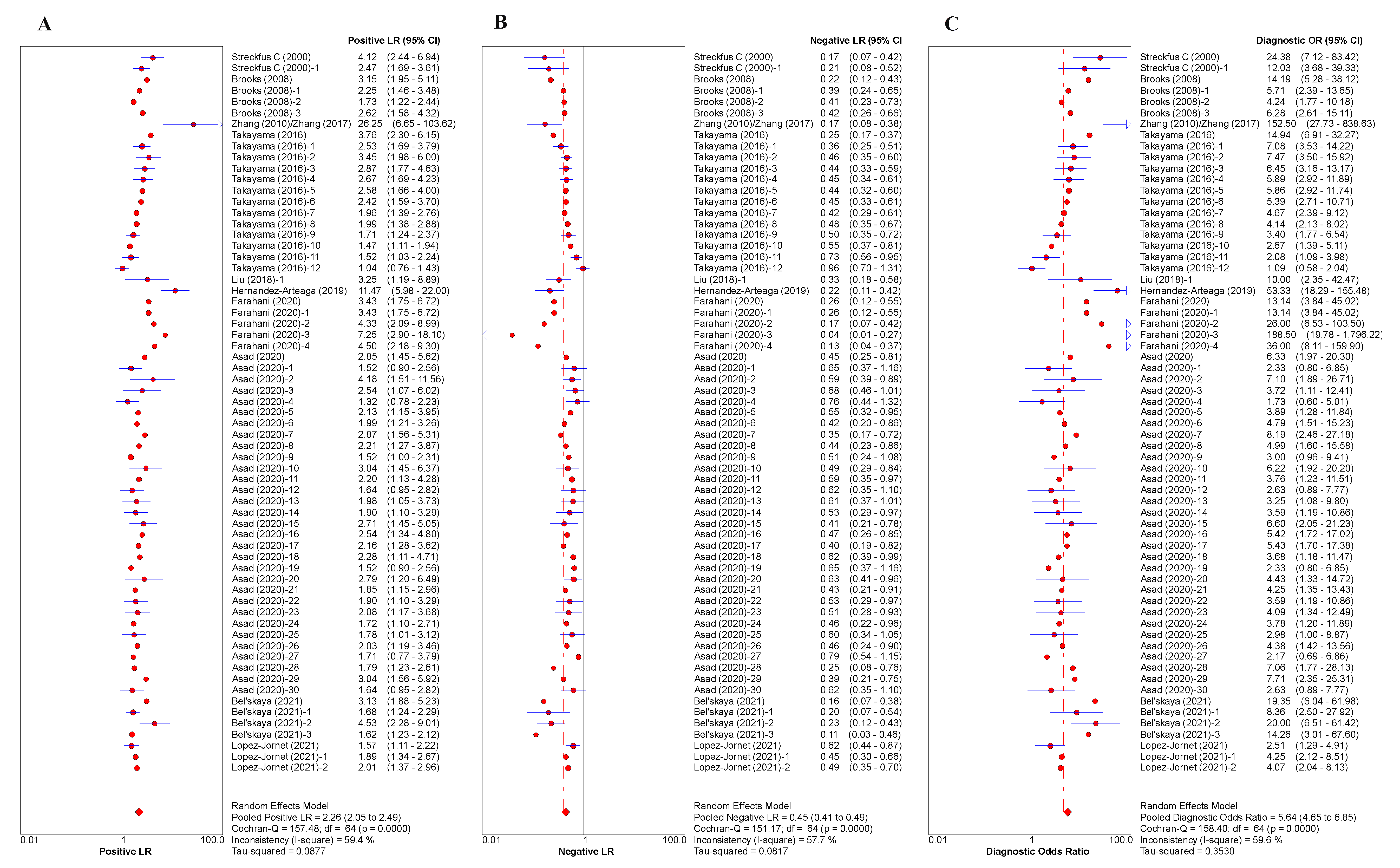


Figure S18. Forest plots of A) PLR, B) NLR, and C) DOR for sample size more than 55 subjects in diagnosis of breast cancer using salivary biomarkers (95% CI).


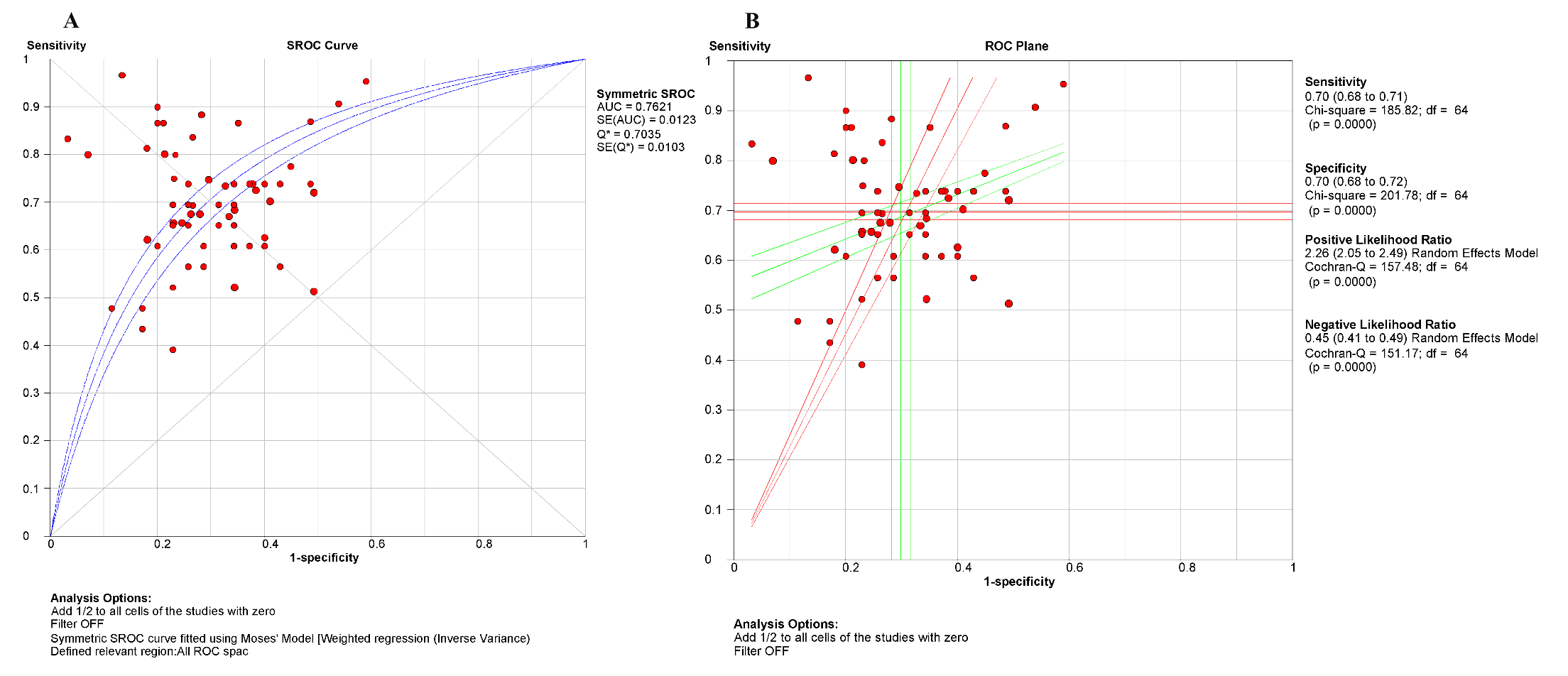


Figure S19. A) ROC plane and B) SROC curve for sample size more than 55 subjects in diagnosis of breast cancer using salivary biomarkers.


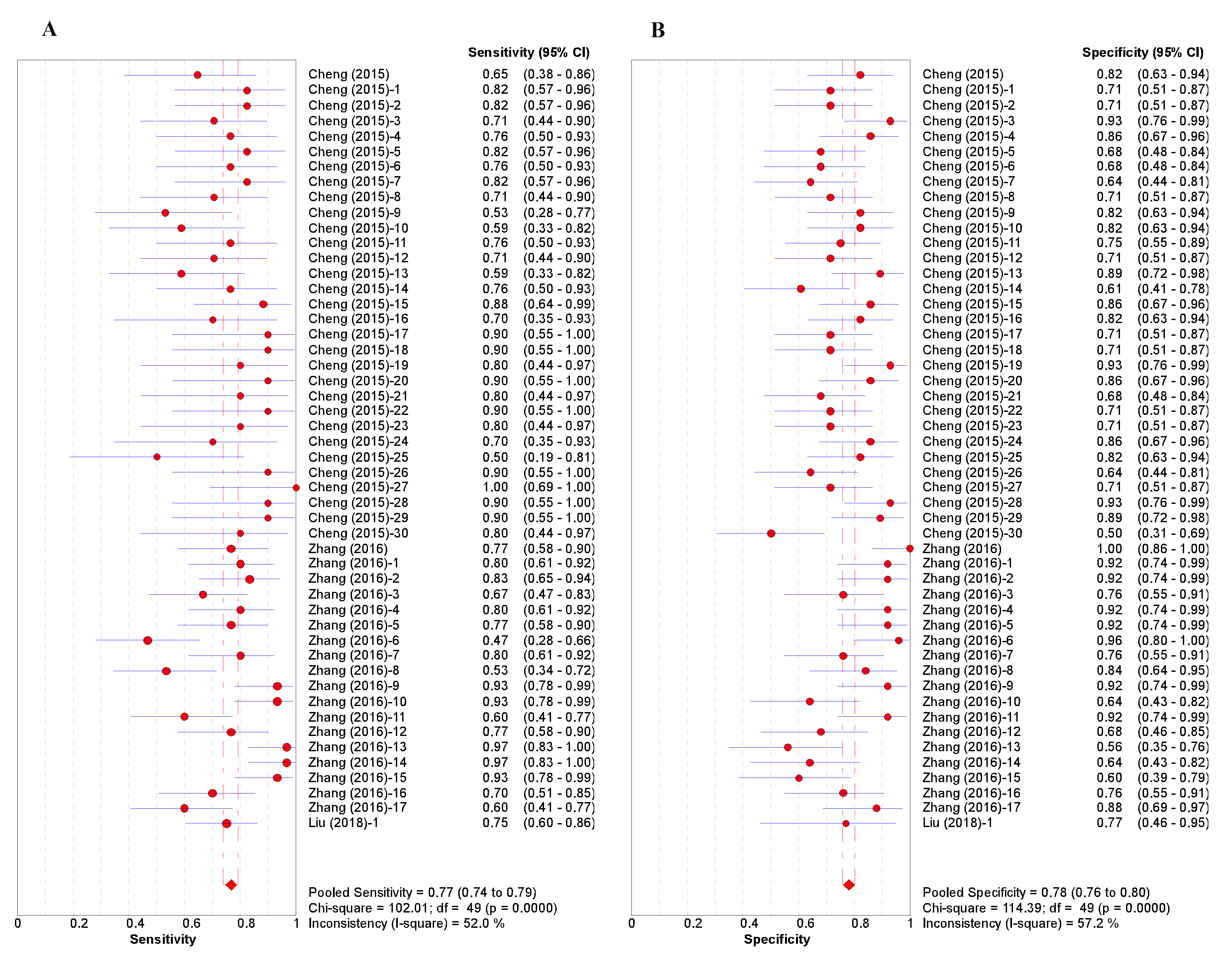


Figure S20. Forest plots of A) sensitivity and B) specificity for diagnosis of breast cancer using salivary biomarkers in Chinese population (95% CI).


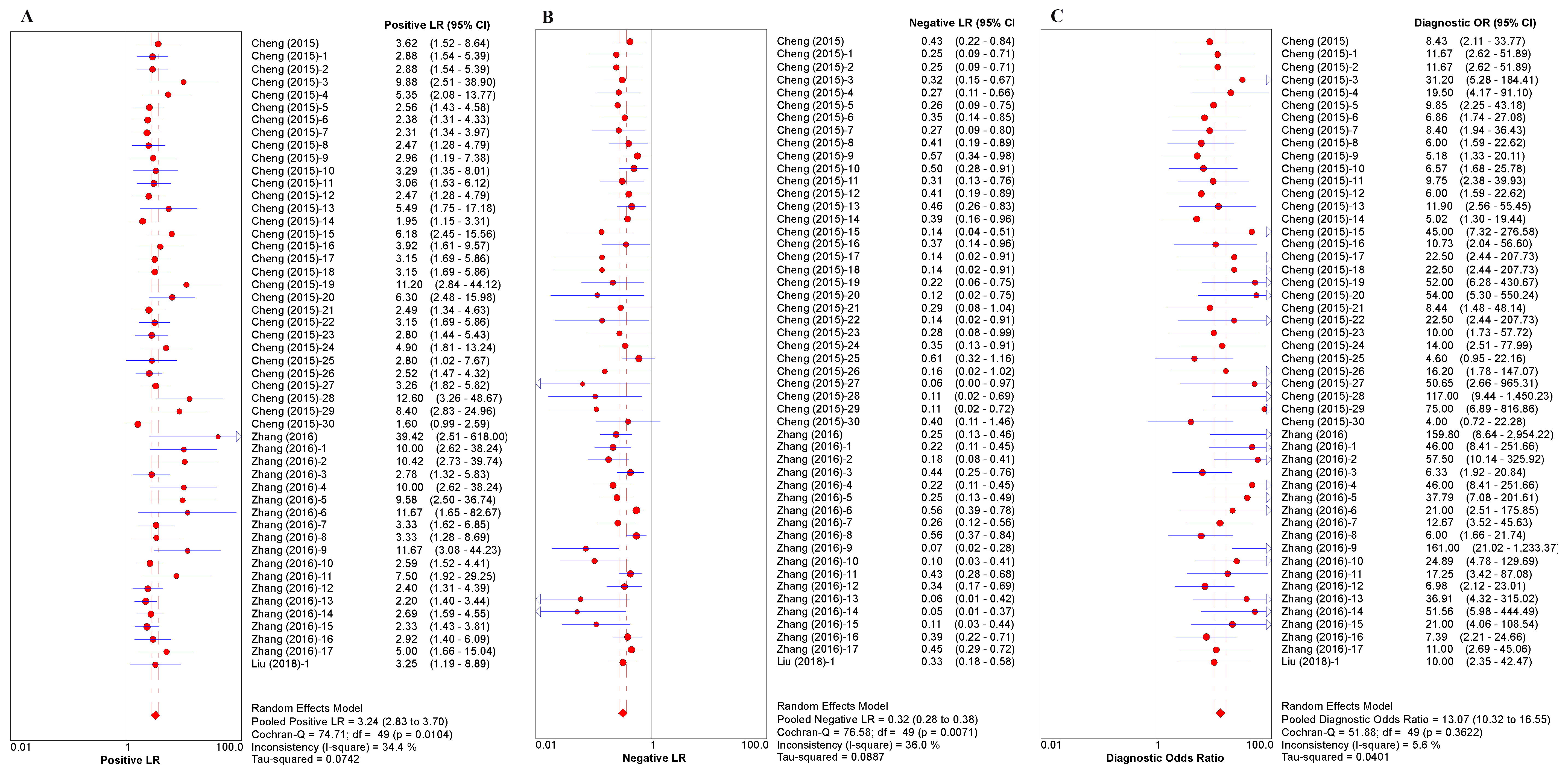


Figure S21. Forest plots of A) PLR, B) NLR, and C) DOR for diagnosis of breast cancer using salivary biomarkers in Chinese population (95% CI).


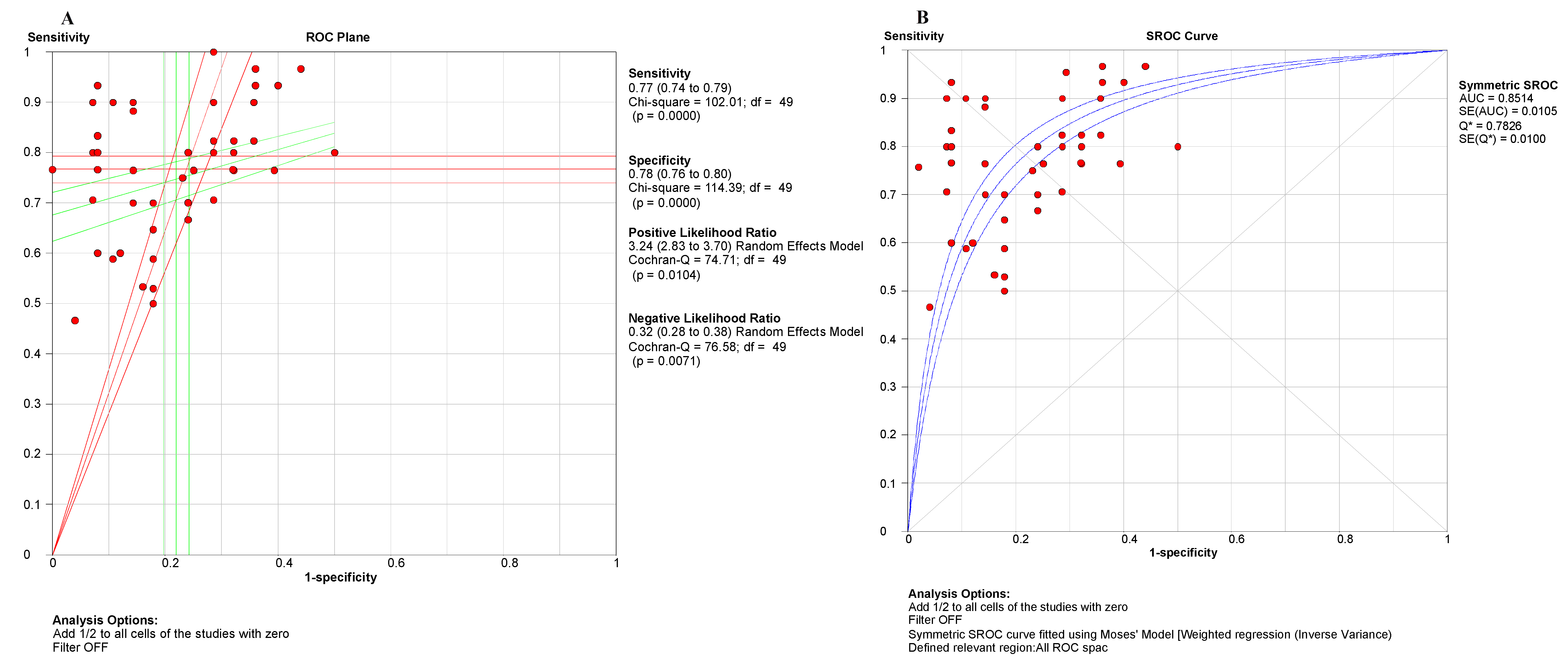


Figure S22. A) ROC plane and B) SROC curve for diagnosis of breast cancer using salivary biomarkers in Chinese population.


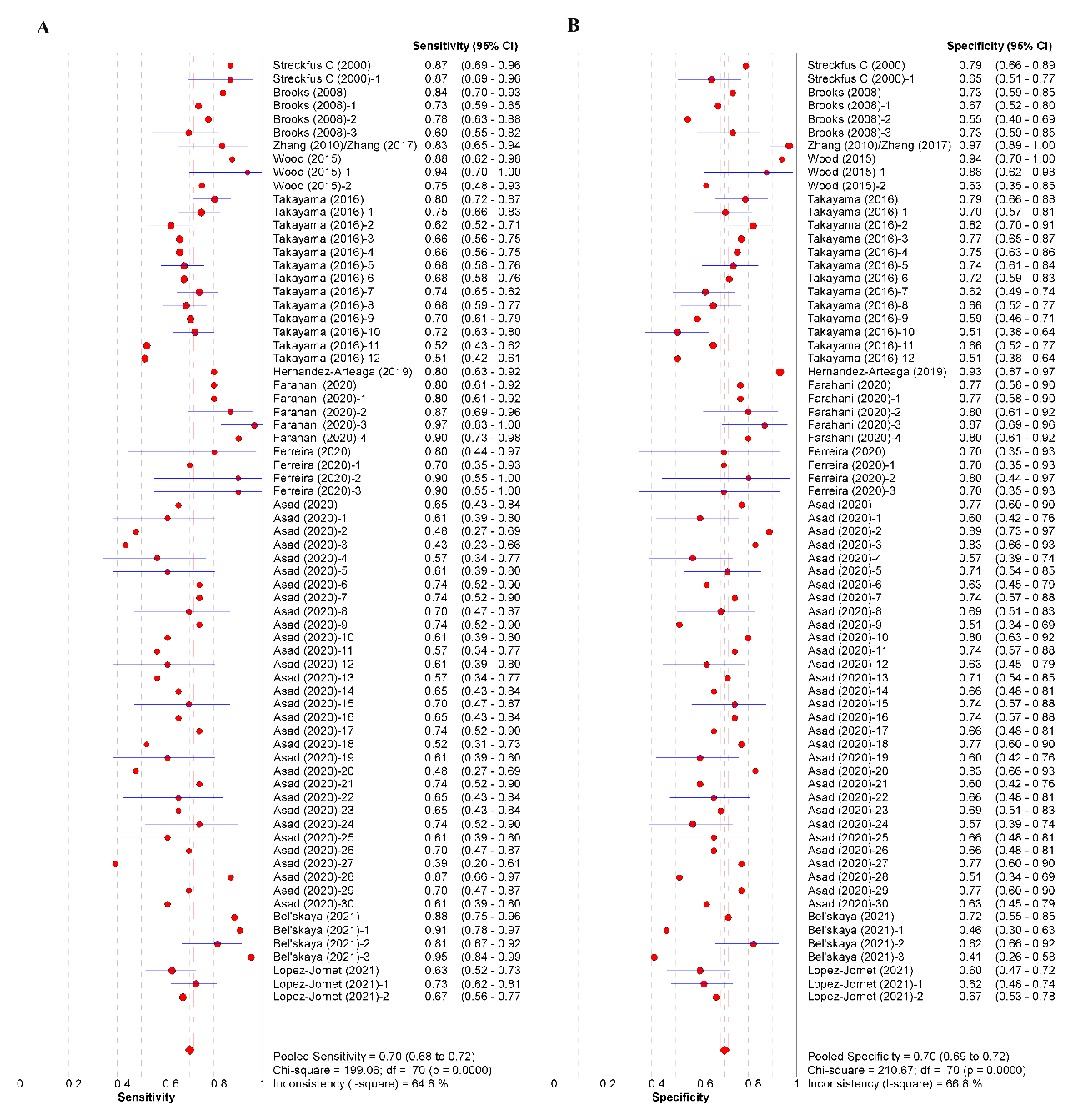


Figure S23. Forest plots of A) sensitivity and B) specificity for diagnosis of breast cancer using salivary biomarkers in non-Chinese population (95% CI).


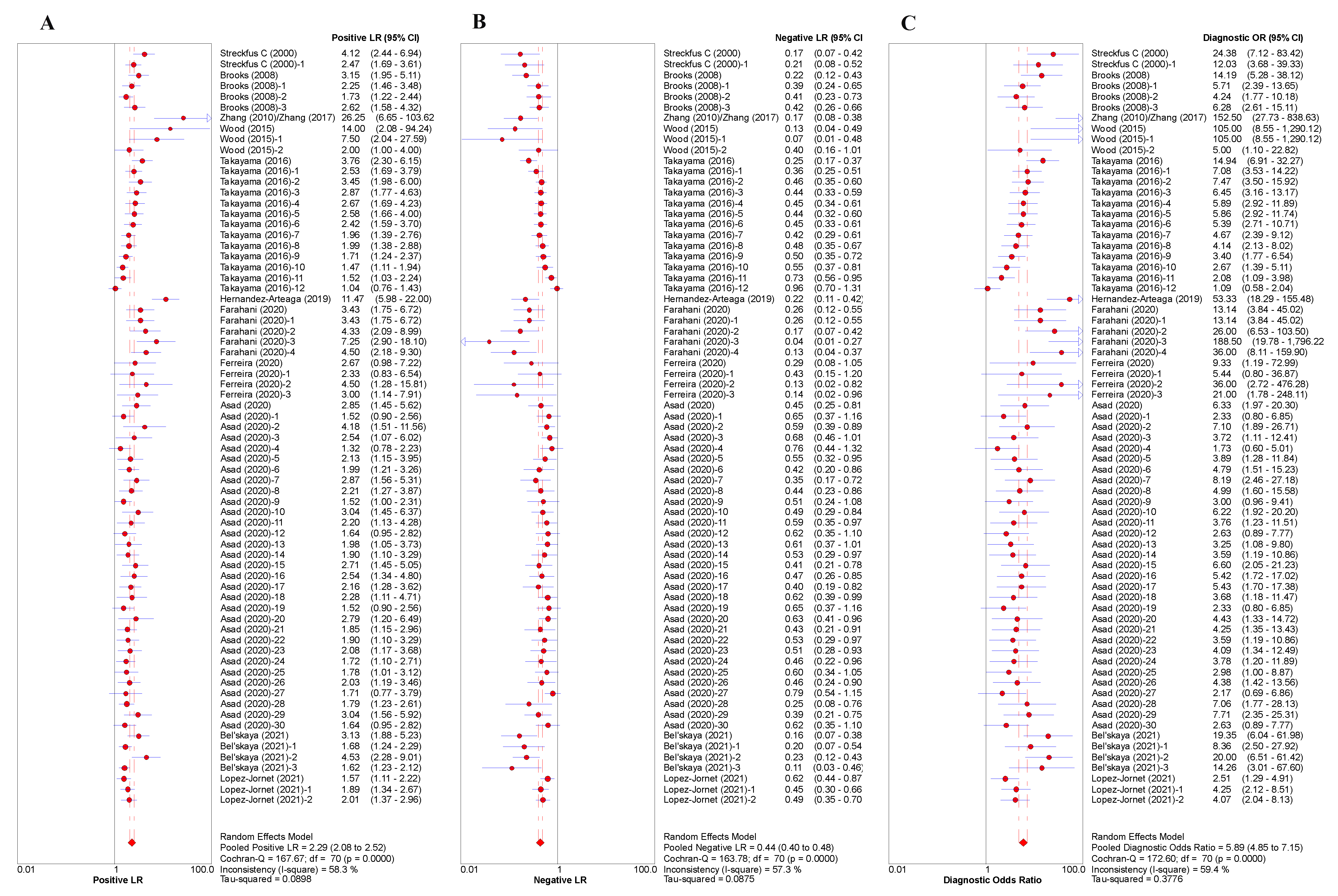


Figure S24. Forest plots of A) PLR, B) NLR, and C) DOR for diagnosis of breast cancer using salivary biomarkers in non-Chinese population (95% CI).


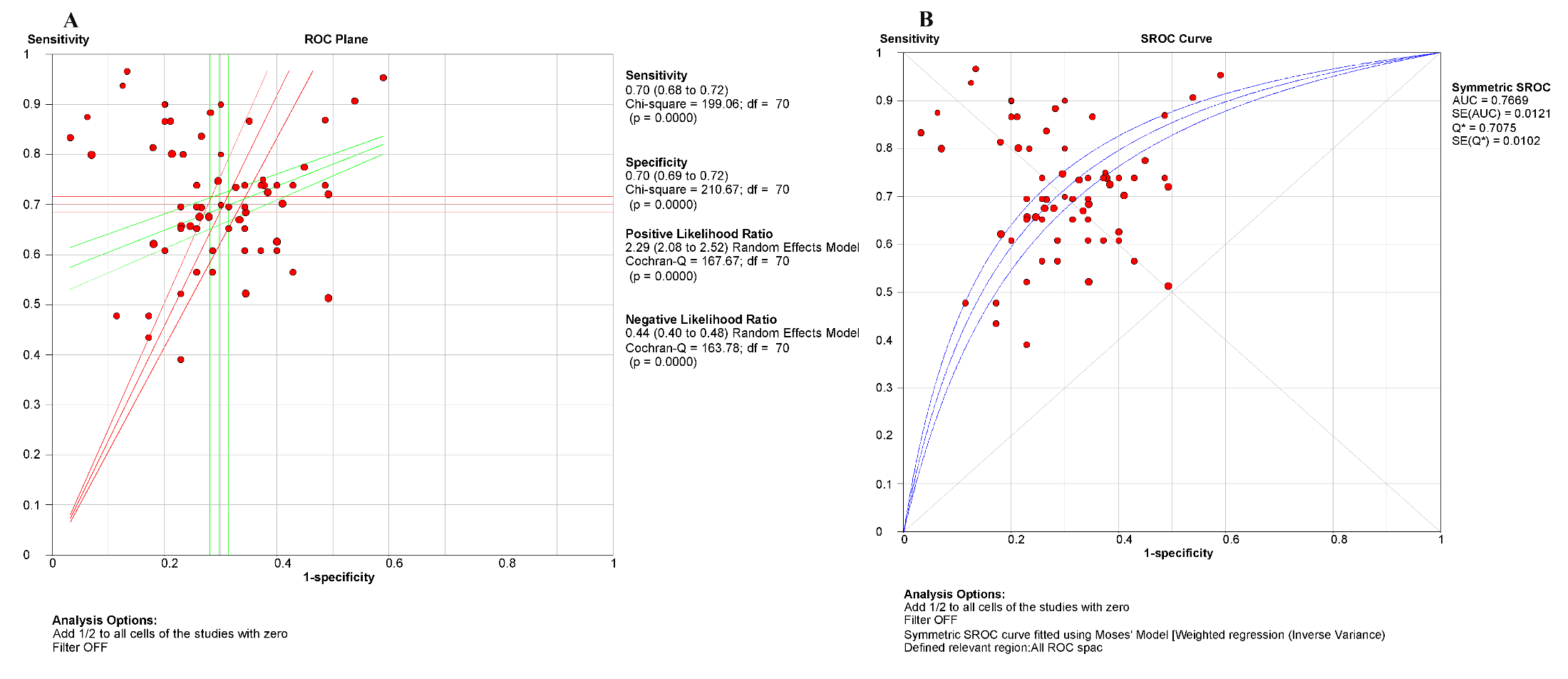


Figure S25. A) ROC plane and B) SROC curve for diagnosis of breast cancer using salivary biomarkers in non-Chinese population.


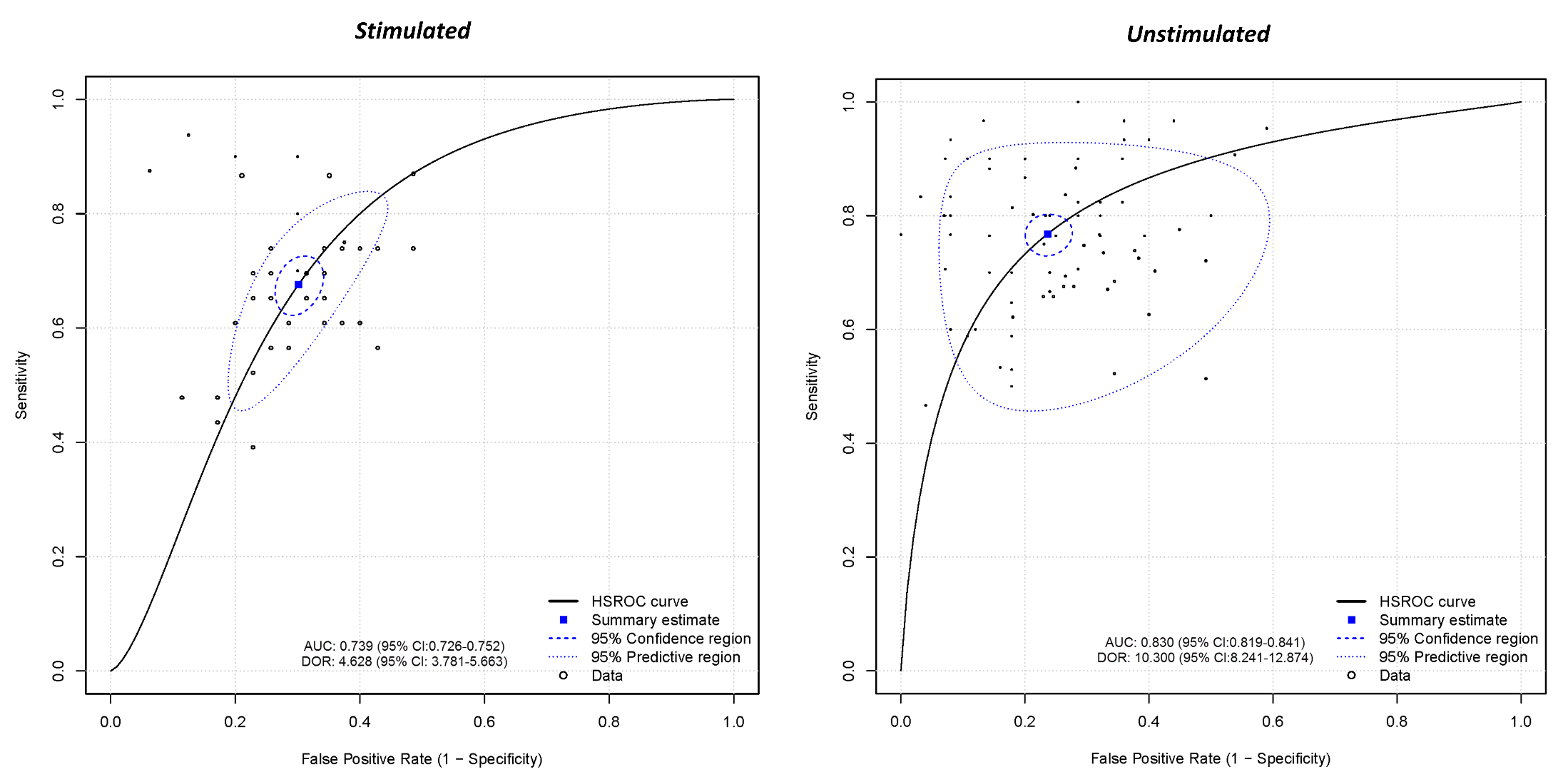


Figure S26. Hierarchical summary receiver operating characteristic (HSROC) curves of subgroup analysis based on saliva type (stimulated & unstimulated) for diagnosis of breast cancer using salivary biomarkers.


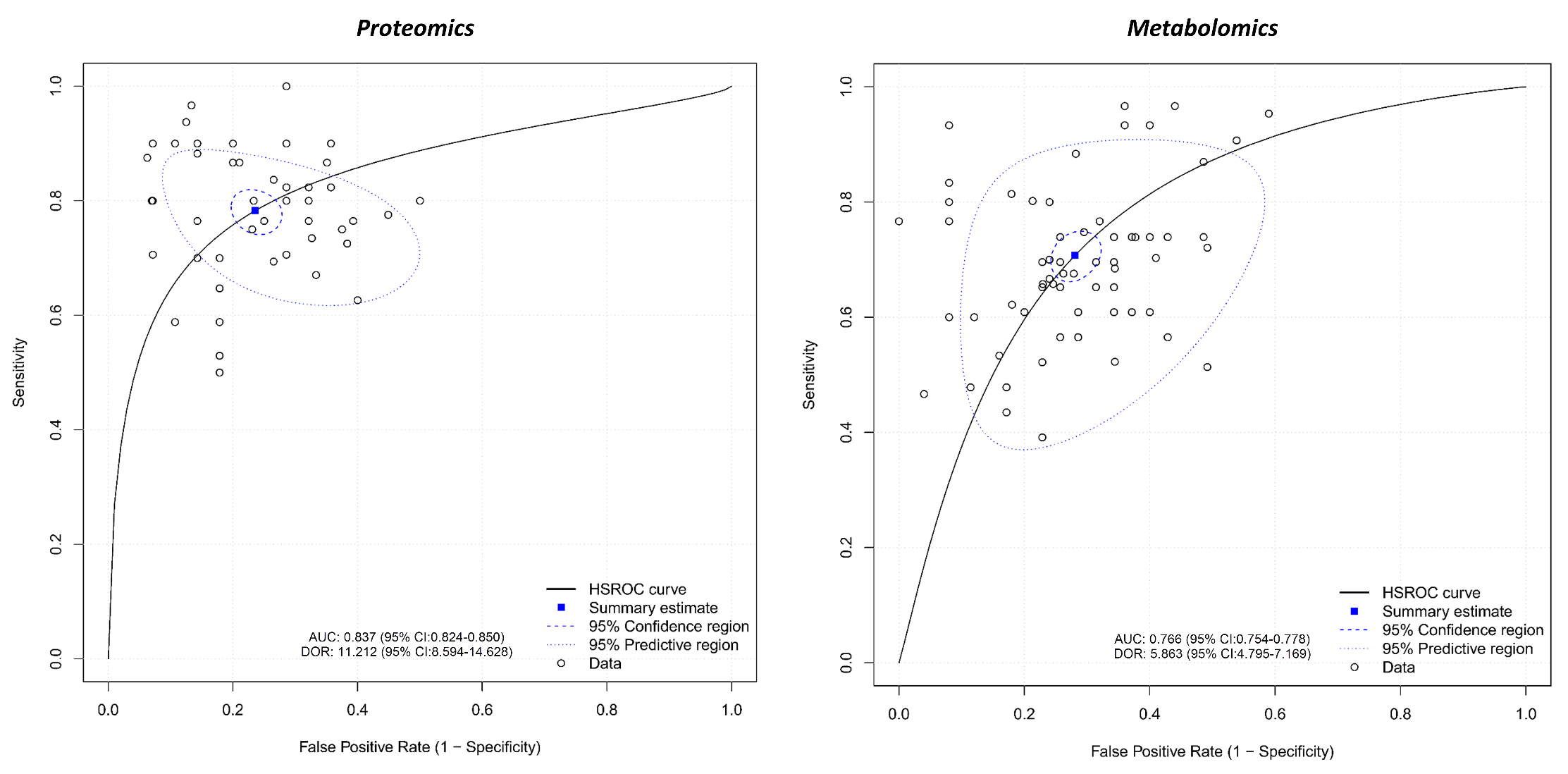
Figure S27. HSROC curves of subgroup analysis based on the saliva type (proteomics and metabolomics) for diagnosis of breast cancer using salivary biomarkers.


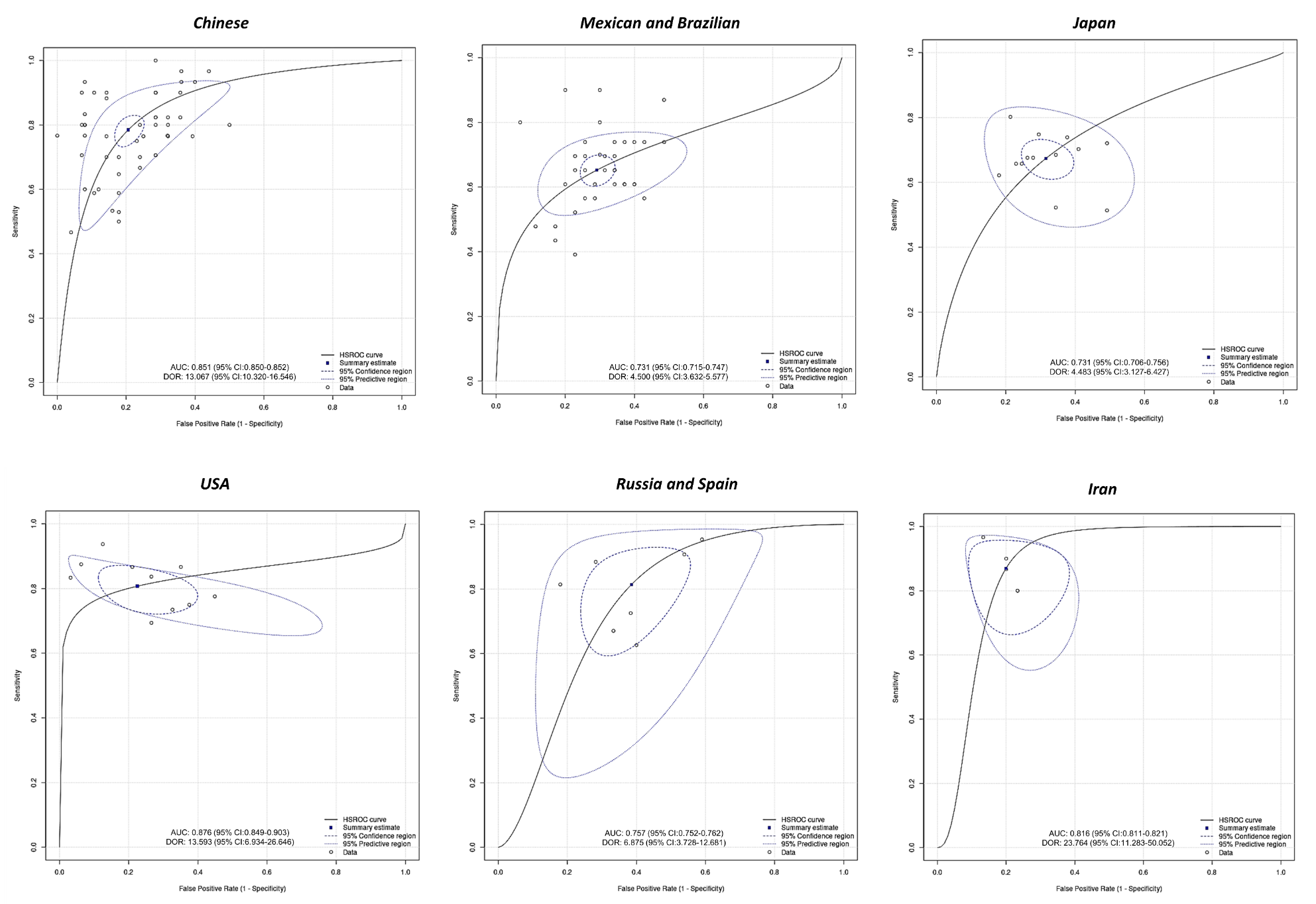


Figure S28. HSROC curves of subgroup analysis based on the ethnicity and countries for diagnosis of breast cancer using salivary biomarkers.

.
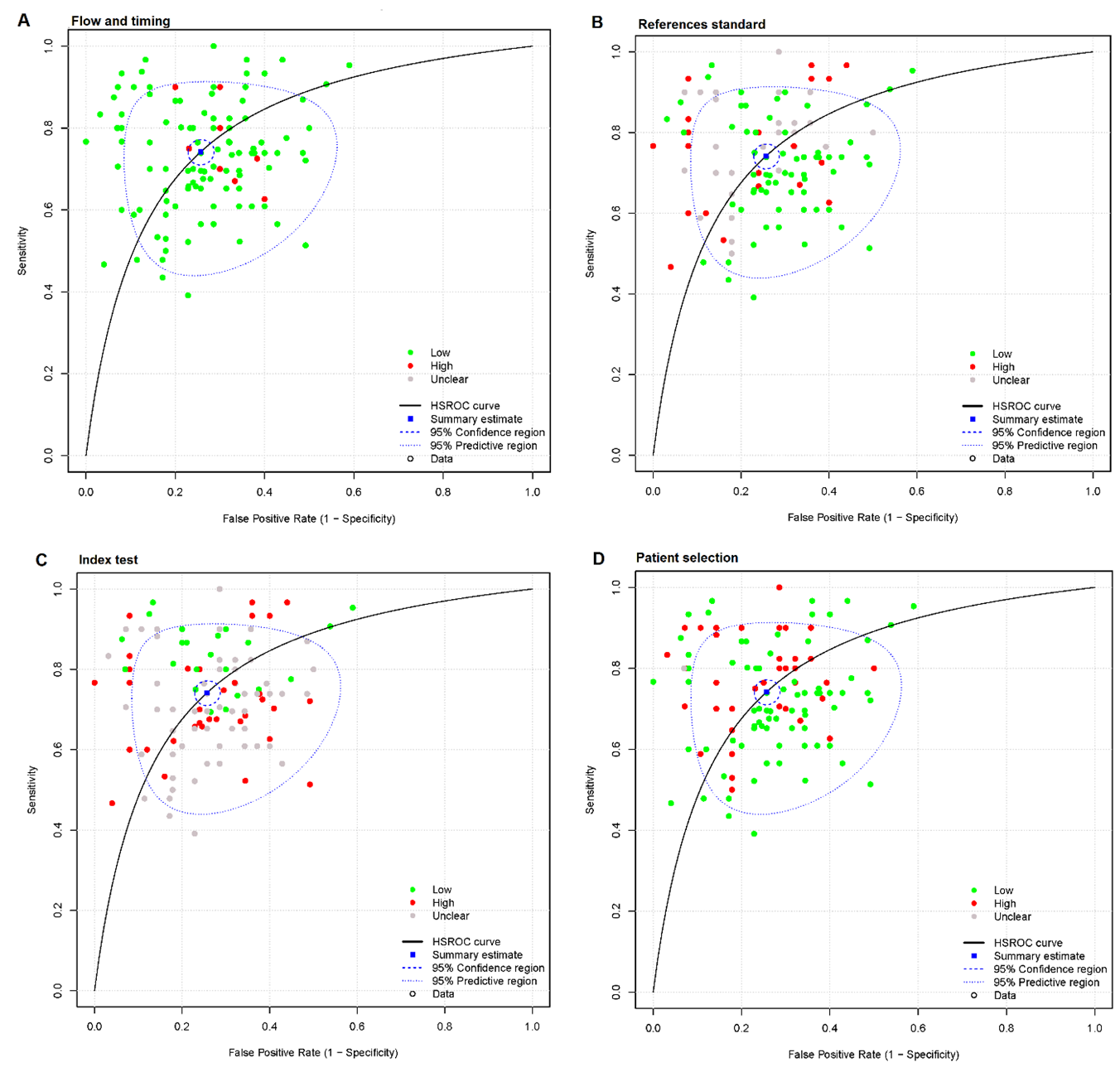


Figure S29. HSROC curves shows the quality assessment scores of each unit study in terms of bias risk, A) flow and timing, B) references standard, C) index test, D) patient selection.


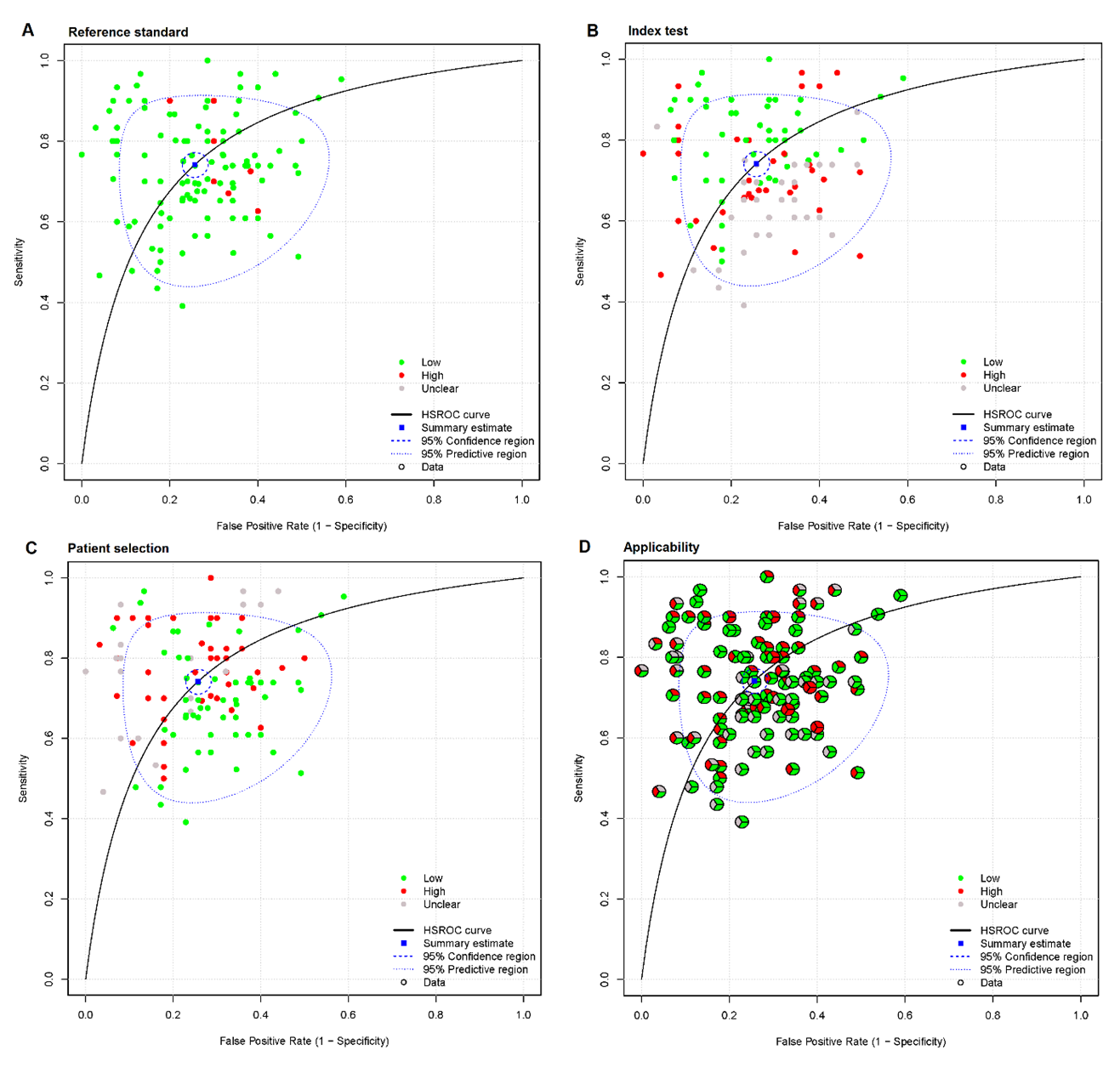


Figure S30. HSROC curves shows the quality assessment scores of each unit study in terms of applicability concern, A) references standard, B) index test, C) patient selection, D) total applicability concern.


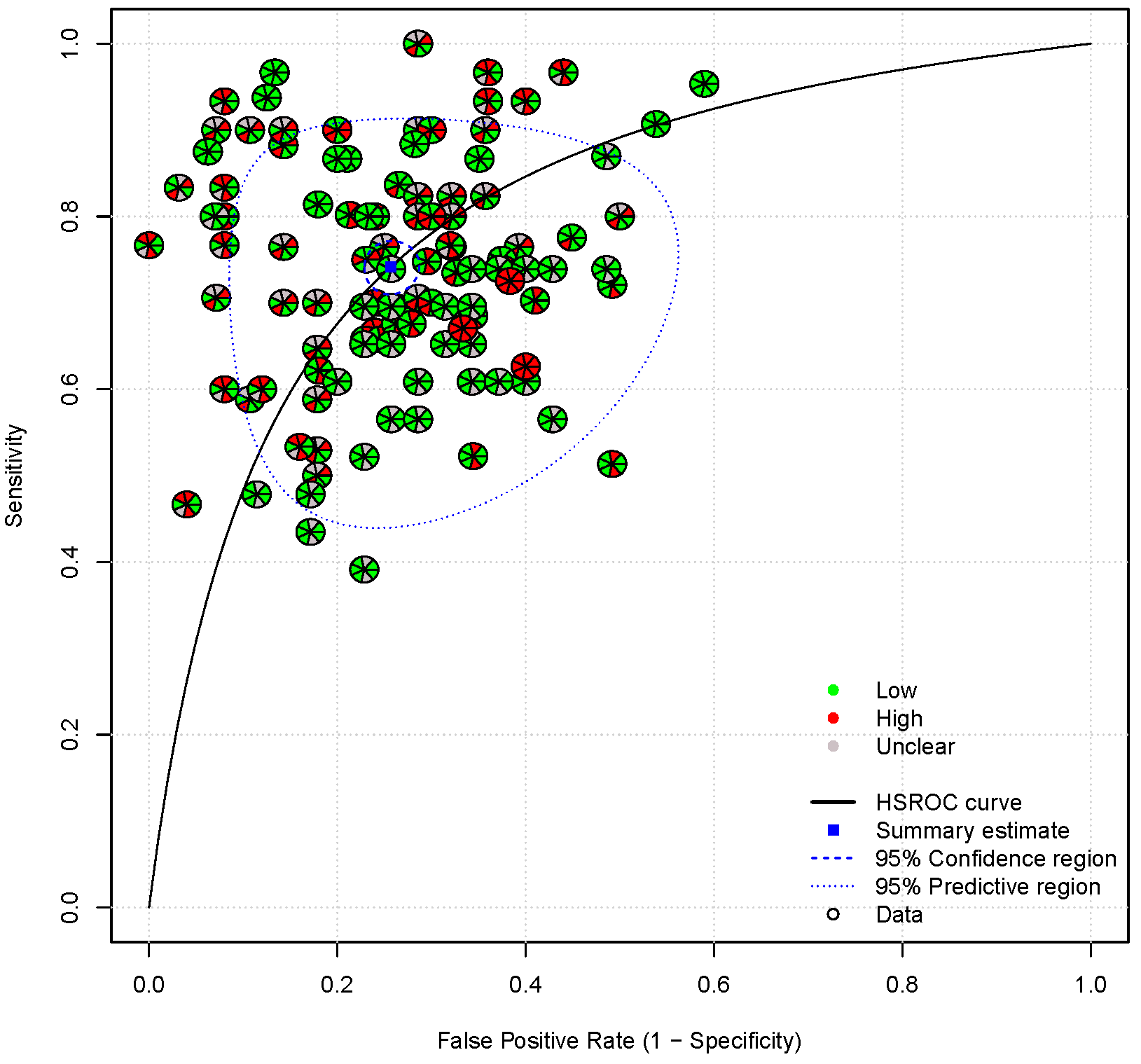


Figure S31. HSROC curves shows the quality assessment scores for each study unit using total risk of bias and applicability concerns.
