# Supplementary material for: Salivary biomarkers in breast cancer diagnosis: A systematic review and diagnostic meta‐analysis
Source: Cancer Med. 2022 Mar 22;11(13):2644–61. doi: 10.1002/cam4.4640 (PMC9249990; doi:10.1002/cam4.4640)
Supplement: Supplementary file 2 — Table S1 Risk of bias assessment and applicability concerns using QUADAS‐2 of included studies. [file CAM4-11-2644-s001.docx]

| Table S1. Risk of bias assessment and applicability concerns using QUADAS-2 of included studies.   \| **Study** \| **RISK OF BIAS** \| \| \| \| **APPLICABILITY CONCERNS** \| \| \| \| --- \| --- \| --- \| --- \| --- \| --- \| --- \| --- \| \| **PATIENT SELECTION** \| **INDEX TEST** \| **REFERENCE STANDARD** \| **FLOW AND TIMING** \| **PATIENT SELECTION** \| **INDEX TEST** \| **REFERENCE STANDARD** \| \| Study 1 \| ☺ \| ☺ \| ☺ \| ☺ \| ☹ \| ☺ \| ☺ \| \| Study 2 \| ☺ \| ☺ \| ☺ \| ☺ \| ☹ \| ☺ \| ☺ \| \| Study 3 \| ☹ \| ☹ \| ☺ \| ☺ \| ☹ \| ☺ \| ☺ \| \| Study 4 \| ☹ \| ☹ \| ☺ \| ☺ \| ☹ \| ☺ \| ☺ \| \| Study 5 \| ☺ \| ☺ \| ☺ \| ☺ \| ☺ \| ☺ \| ☺ \| \| Study 6 \| ☺ \| ☹ \| ☹ \| ☺ \| ? \| ☹ \| ☺ \| \| Study 7 \| ☺ \| ☹ \| ☺ \| ☺ \| ☺ \| ☹ \| ☺ \| \| Study 8 \| ☹ \| ☺ \| ☺ \| ☹ \| ☺ \| ? \| ☺ \| \| Study 9 \| ? \| ☺ \| ☺ \| ☺ \| ? \| ☺ \| ☺ \| \| Study 10 \| ☺ \| ☺ \| ☺ \| ☺ \| ☺ \| ☺ \| ☺ \| \| Study 11 \| ☹ \| ☺ \| ☺ \| ☹ \| ☹ \| ☺ \| ☹ \| \| Study 12 \| ☺ \| ? \| ☺ \| ☺ \| ☺ \| ? \| ☺ \| \| Study 13 \| ☺ \| ☺ \| ☺ \| ☺ \| ☺ \| ☺ \| ☺ \| \| Study 14 \| ☹ \| ☹ \| ☹ \| ☹ \| ☹ \| ☹ \| ☹ \|  \| ☺ Low risk \| ☹ High risk \| ? Unclear \| \| --- \| --- \| --- \| |
| --- | --- | --- | --- | --- | --- | --- | --- | --- | --- | --- | --- | --- | --- | --- | --- | --- | --- | --- | --- | --- | --- | --- | --- | --- | --- | --- | --- | --- | --- | --- | --- | --- | --- | --- | --- | --- | --- | --- | --- | --- | --- | --- | --- | --- | --- | --- | --- | --- | --- | --- | --- | --- | --- | --- | --- | --- | --- | --- | --- | --- | --- | --- | --- | --- | --- | --- | --- | --- | --- | --- | --- | --- | --- | --- | --- | --- | --- | --- | --- | --- | --- | --- | --- | --- | --- | --- | --- | --- | --- | --- | --- | --- | --- | --- | --- | --- | --- | --- | --- | --- | --- | --- | --- | --- | --- | --- | --- | --- | --- | --- | --- | --- | --- | --- | --- | --- | --- | --- | --- | --- | --- | --- | --- | --- | --- | --- | --- | --- | --- | --- |
|  |
