# Supplementary material for: Salivary biomarkers in breast cancer diagnosis: A systematic review and diagnostic meta‐analysis
Source: Cancer Med. 2022 Mar 22;11(13):2644–61. doi: 10.1002/cam4.4640 (PMC9249990; doi:10.1002/cam4.4640)
Supplement: Supplementary file 3 — Table S2 Subgroup analysis of saliva for breast cancer diagnosis based on different covariates. [file CAM4-11-2644-s002.docx]

Table S2. Subgroup analysis of saliva for breast cancer diagnosis based on different covariates.

|  | **Study units** | **DOR (95% CI)** | **I^2^ (%)** | **AUC (95% CI)** | **Sensitivity (95% CI)** | **Specificity (95% CI)** | **PLR (95% CI)** | **NLR (95% CI)** | $\boldsymbol{\tau}^{\boldsymbol{2}}$ | **Z-value** | **Cochran-Q** |
| --- | --- | --- | --- | --- | --- | --- | --- | --- | --- | --- | --- |
| **All** | 121 | 7.837 (6.624-9.277) | 55.991 | 0.801 (0.800-0.802) | 0.717 (0.703-0.730) | 0.727 (0.713-0.740) | 2.597 (2.389-2.824) | 0.396 (0.364-0.432) | 0.444 | 34.332 | 272.67 |
| **Mean age of patients** |  |  |  |  |  |  |  |  |  |  |  |
| ***Equal or less than 52 years old (age≤52)*** | 74 | 6.092 (5.122-7.246) | 24.10 | 0.770 (0.760-0.780) | 0.711 (0.689-0.733) | 0.708 (0.689-0.726) | 2.348 (2.157-2.557) | 0.446 (0.404-0.492) | 0.132 | 220.445 | 96.14 |
| ***More than 52 years old (age>50)*** | 47 | 11.212 (8.175-15.376) | 73.53 | 0.840 (0.824-0.856) | 0.720 (0.702-0.738) | 0.752 (0.732-0.772) | 3.068 (2.595-3.627) | 0.345 (0.298-0.399) | 0.772 | 15.022 | 173.81 |
| **Saliva type** | | | | | | | | | | | |
| ***Unstimulated*** | 81 | 10.300 (8.241-12.874) | 63.30 | 0.830 (0.819–0.841) | 0.729 (0.713–0.744) | 0.740 (0.724–0.755) | 2.912 (2.600–3.261) | 0.350 (0.313–0.391) | 0.564 | 30.894 | 216.98 |
| ***Stimulated*** | 40 | 4.628 (3.781–5.663) | 7.30 | 0.739 (0.726-0.752) | 0.670 (0.638–0.702) | 0.697 (0.671–0.722) | 2.094 (1.906–2.301) | 0.516 (0.463–0.576) | 0.030 | 15.428 | 41.96 |
| **Biomarker measurement method** | | | | | | | | | | | |
| ***Mass spectrometry*** | 93 | 6.663 (5.578–7.958) | 48.60 | 0.781 (0.771–0.791) | 0.692 (0.675–0.708) | 0.724 (0.708–0.740) | 2.480 (2.269–2.710) | 0.439 (0.403–0.479) | 0.325 | 28.271 | 178.22 |
| *UPLC–MS* | 31 | 3.947 (3.215-4.847) | <0.0001 | 0.715 (0.700-0.730) | 0.633 (0.596-0.668) | 0.688 (0.660-0.716) | 1.967 (1.776-2.178) | 0.559 (0.506-0.619) | <0.0001 | 13.104 | 13.91 |
| *UPLC–ESI–MS* | 31 | 10.961 (8.148-14.745) | <0.0001 | 0.836 (0.821-0.851) | 0.765 (0.722-0.805) | 0.765 (0.735-0.793) | 3.049 (2.634-3.530) | 0.361 (0.305-0.427) | <0.0001 | 15.822 | 24.43 |
| *HILIC-UPLC-ESI-MS* | 31 | 8.674 (6.100-12.334) | 72.70 | 0.815 (0.795-0.835) | 0.697 (0.677-0.718) | 0.727 (0.702-0.752) | 2.697 (2.241-3.246) | 0.395 (0.339-0.460) | 0.617 | 20.201 | 109.78 |
| ***Non-mass spectrometry*** | 28 | 12.924 (8.639-19.332) | 66.30 | 0.859 (0.669-0.878) | 0.790 (0.764-0.814) | 0.735 (0.707-0.761) | 3.000 (2.442-3.685) | 0.272 (0.218-0.339) | 0.692 | 19.850 | 79.87 |
| *ELISA* | 15 | 12.754 (8.681–18.739) | 38.00 | 0.845 (0.820–0.870) | 0.837 (0.805–0.867) | 0.695 (0.657–0.731) | 2.814 (2.245–3.526) | 0.245 (0.191–0.314) | 0.211 | 16.244 | 22.54 |
| Western blot | 4 | 20.044 (4.694-85.593) | 56.90 | 0.899 (0.838-0.960) | 0.802 (0.708-0.876) | 0.803 (0.682-0.894) | 4.027 (1.730-9.373) | 0.249 (0.126-0.491) | 0.199 | 5.794 | 6.93 |
| *ATR-FTIR Spectroscopy* | 4 | 11.727 (3.897-35.297) | 0.00 | 0.7684 (0.662-0.874) | 0.825 (0.672-0.927) | 0.725 (0.561-0.854) | 2.920 (1.730-4.929) | 0.280 (0.140-0.559) | <0.0001 | 4.379 | 1.60 |
| *Immunoassays* | 3 | 3.486 (2.347-5.177) | 0.00 | 0.684 (0.638-0.730) | 0.674 (0.615-0.729) | 0.628 (0.553-0.699) | 1.801 (1.465-2.215) | 0.523 (0.426-0.642) | <0.0001 | 6.190 | 1.42 |
| *RT-qPCR* | 1 | 152.50 (27.73–838.624) | 0.00 | ---- | 0.830 (0.655–0.945) | 0.97 (0.895–1.000) | 26.25 (6.65–103.62) | 0.170 (0.08–0.38) | <0.0001 | 5.780 | <0.0001 |
| *SERS* | 1 | 53.330 (18.290-155.480) | 0.00 | 0.950 | 0.800 (0.630-0.920) | 0.93 (0.870-0.970) | 11.470 (5.980-22.000) | 0.220 (0.110-0.420) | <0.0001 | 7.284 | <0.0001 |
| **Sample size** | | | | | | | | | | | |
| ***>55*** | 65 | 5.643 (4.649-6.849) | 59.60 | 0.762 (0.750-0.774) | 0.697 (0.681-0.714) | 0.701 (0.684-0.718) | 2.262 (2.051-2.494) | 0.447 (0.405-0.493) | 0.353 | 26.804 | 158.40 |
| ***≤55*** | 56 | 13.395 (10.596-16.932) | 8.70 | 0.853 (0.843-0.863) | 0.773 (0.747-0.798) | 0.780 (0.758-0.802) | 3.230 (2.836-3.678) | 0.315 (0.272-0.366) | 0.069 | 22.690 | 60.27 |
| **Biomarker type** | |  | |  |  |  |  |  |  |  |  |
| ***Metabolomic*** | 65 | 5.863 (4.795–7.169) | 55.10 | 0.766 (0.754–0.778) | 0.689 (0.671–0.706) | 0.707 (0.689–0.725) | 2.273 (2.056–2.512) | 0.452 (0.410–0.498) | 0.342 | 25.000 | 141.95 |
| ***Proteomic*** | 51 | 11.212(8.594-14.628) | 44.50 | 0.837 (0.824–0.850) | 0.772 (0.748–0.795) | 0.746 (0.725–0.766) | 3.025 (2.641–3.465) | 0.322 (0.278–0.373) | 0.354 | 23.245 | 89.91 |
| ***Reagent-free biophotonic*** | 4 | 11.727 (3.897-35.297) | 0.000 | 0.768 (0.663–0.873) | 0.825 (0.672–0.927) | 0.725 (0.561–0.854) | 2.920 (1.730–4.929) | 0.280 (0.140–0.559) | <0.0001 | 4.379 | 1.60 |
| ***Transcriptomic & Proteomic*** | 1 | 152.50 (27.73–838.624) | 0.000 | - | 0.830 (0.655–0.945) | 0.97 (0.895–1.000) | 26.25 (6.65–103.62) | 0.170 (0.08–0.38) | <0.0001 | 5.780 | <0.0001 |
| **Type of control** |  |  |  |  |  |  |  |  |  |  |  |
| ***HC*** | 112 | 7.272 (6.126–8.632) | 54.2 | 0.87 (0.86–0.88) | 0.707 (0.692–0.721) | 0.722 (0.707–0.736) | 2.520 (2.316–2.741) | 0.416 (0.382–0.452) | 0.404 | 22.914 | 240.29 |
| ***BC & HC*** | 9 | 17.533 (11.247–27.334) | 5.00 | 0.92 (0.89–0.94) | 0.836 (0.788–0.876) | 0.774 (0.729–0.814) | 3.373 (2.241–5.079) | 0.233 (0.178–0.306) | 0.023 | 13.197 | <0.0001 |
| **Nations** |  |  |  |  |  |  |  |  |  |  |  |
| ***Non-Chinese*** | 71 | 5.888 (4.848-7.150) | 59.4 | 0.767 (0.755-0.779) | 0.701 (0.684-0.717) | 0.703 (0.686-0.719) | 2.292 (2.081-2.525) | 0.439 (0.398-0.485) | 0.378 | 11.349 | 108.44 |
| ***Chinese*** | 50 | 13.067 (10.320-16.546) | 5.6 | 0.851 (0.850-0.852) | 0.766 (0.739-0.792) | 0.781 (0.757-0.803) | 3.235 (2.827-3.702) | 0.324 (0.279-0.376) | 0.040 | 20.726 | 59.47 |
| **Ethnicity & Countries** | | | | | | | | | | | |
| ***Chinese*** | 50 | 13.067 (10.320-16.546) | 5.6 | 0.851 (0.850-0.852) | 0.766 (0.739-0.792) | 0.781 (0.757-0.803) | 3.235 (2.827-3.702) | 0.324 (0.279-0.376) | 0.040 | 20.726 | 59.47 |
| ***Mexico and Brazil*** | 36 | 4.500 (3.632-5.577) | 13.60 | 0.731 (0.714-0.747) | 0.650 (0.615-0.683) | 0.715 (0.689-0.739) | 2.131 (1.889-2.404) | 0.540 (0.489-0.571) | 0.058 | 13.104 | 13.91 |
| ***Japan*** | 13 | 4.483 (3.127-6.427) | 72.2 | 0.731 (0.706-0.756) | 0.671 (0.646-0.695) | 0.680 (0.646-0.712) | 2.102 (1.712-2.582) | 0.481 (0.406-0.569) | 0.316 | 15.022 | 43.19 |
| ***USA*** | 10 | 13.593 (6.934-26.646) | 66.0 | 0.876 (0.849-0.903) | 0.799 (0.752-0.842) | 0.746 (0.701-0.787) | 3.081 (2.193-4.327) | 0.280 (0.207-0.380) | 0.709 | 12.288 | 26.31 |
| ***Russia and Spain*** | 7 | 6.785 (3.728-12.681) | 66.7 | 0.757 (0.707-0.807) | 0.757 (0.715-0.796) | 0.616 (0.562-0.668) | 1.984 (1.595-2.467) | 0.334 (0.223-0.501) | 0.427 | 9.851 | 17.96 |
| ***Iran*** | 4 | 23.764 (141.283-50.052) | 25.2 | 0.816 (0.811-0.821) | 0.867 (0.802-0.917) | 0.800 (0.727-0.861) | 4.176 (3.013-5.787) | 0.188 (0.115-0.307) | 0.182 | 75.076 | 4.31 |
|  |  |  |  |  |  |  |  |  |  |  |  |

CI: confidence interval; PLR: positive likelihood ratio; NLR: negative likelihood ratio; DOR: diagnostic odds ratio; AUC: area under the ROC curve, HC: healthy controls; BC: benign controls.
